# Strong Chiro-Optical Activity of Plasmonic Metasurfaces with Inverted Pyramid Arrays

**DOI:** 10.1021/acsami.4c19803

**Published:** 2025-03-03

**Authors:** Luis Alberto Pérez, Jinhui Hu, Jose Mendoza-Carreño, Miquel Garriga, Maria Isabel Alonso, Oriol Arteaga, Alejandro R. Goñi, Agustín Mihi

**Affiliations:** † Institute of Materials Science of Barcelona, ICMAB-CSIC, Campus de la UAB, 08193 Bellaterra, Catalonia, Spain; ‡ Department of Applied Physics, PLAT Group, University of Barcelona, 08028 Barcelona, Spain; § ICREA, Passeig Lluís Companys 23, 08010 Barcelona, Spain

**Keywords:** chiral metasurfaces, high g-factors, plasmonics, silicon etching, inverted pyramids

## Abstract

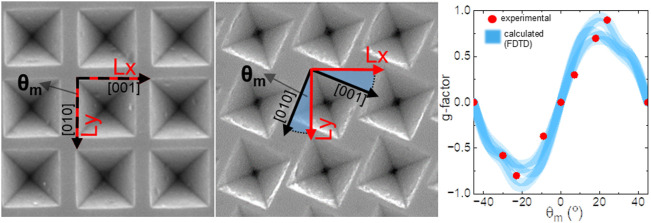

Chiral plasmonics has emerged as a powerful tool for manipulating light at the nanoscale with unprecedented control over light polarization. The advances in nanofabrication have led to the creation of nanostructures that support strong chiroptical responses. However, the complexity of the fabrication and the associated high costs remain major challenges in upscaling these architectures. Here, we report on the development of chiral plasmonic metasurfaces composed of inverted pyramid arrays with mismatched directions with respect to the lattice vectors of the array. These metasurfaces are fabricated using a combination of soft lithography and anisotropic etching, resulting in cost-effective and reproducible chiral nanostructures without the need for expensive equipment. The fabricated metasurfaces exhibit high differential transmittance values in the visible spectrum, which are among the highest reported for plasmonic films. Theoretical modeling corroborates the experimental results, demonstrating the significant influence of the mismatch angle on the chiral behavior. Complete polarimetric characterization reveals exceptional chiro-optical activity with circular birefringence exceeding 375°/μm and Kuhn’s dissymmetry factors (g-factors) approaching unity.

## Introduction

Chirality is a fascinating phenomenon existing on different scales of nature. In chemistry, chiral molecules can be distinguished optically due to the differential interaction of matter with circularly polarized light (CPL). This selective response is prevalent in many natural biomolecules presenting chiral centers that allow the existence of molecules with the exact chemical composition but different symmetries (enantiomers/enantiomorphs). The significance of chiral phenomena transcends disciplinary boundaries, impacting fields such as biochemistry and pharmacy, where it is observed that each molecular enantiomer can exhibit diametrically opposed biological behavior.^1^

The two main observables of chiral phenomena are circular dichroism (CD) and optical rotary dispersion (ORD), also called circular birefringence (CB). These magnitudes represent the differences in the extinction coefficients and refractive indices depending on the handedness of the circularly polarized light. While CD induces a change in the ellipticity of the incident light, ORD preserves the linear polarization state of light but rotates it by a certain azimuthal angle. CD and ORD are Kramers–Kronig related,^2^ giving rise to the so-called Cotton effect that describes the characteristic change in CD and ORD near an absorption band of a chiral molecule, producing distinct positive or negative peaks in the spectrum.^3,4^ Naturally occurring chiral molecules typically exhibit weak chiro-optical responses, which are often in the ultraviolet (UV) region.^5^

Chiral nanophotonics has become a key area for manipulating light on the nanoscale. Advances in nanofabrication have enabled the creation of artificial chiral nanostructures with significantly enhanced chiro-optical activity, allowing for selective interaction with circularly polarized light across specific spectral regions.^6−8^ This ability arises from the interplay between the spatial symmetry of chiral nanostructures and the electromagnetic field, a phenomenon that has been extensively explored in recent studies of chiral metasurfaces and metamaterials.^6,7,9−15^ Notably, such systems provide unprecedented control over light polarization and optical activity, paving the way for applications in areas like optical sensing, where chirality enhances detection sensitivity^16^ and asymmetric catalysis, where selective interactions between chiral molecules and light can influence reaction pathways,^17^ and chiro-optical communication, which exploits the polarization-dependent properties of chiral materials to encode and process information.^18^

By bridging the concepts of plasmonics and chirality, recent literature has demonstrated how these systems can serve as a versatile platform for achieving tunable and polarization-sensitive optical responses.^19−21^ Chiral plasmonics leverages the intrinsic handedness of chiral structures to interact with circularly polarized light, enabling the excitation of distinct (localized) surface plasmon resonances ((L)SPRs) depending on the polarization state. For instance, investigations into chiral plasmonic systems on metasurfaces have revealed how nanoscale fabrication techniques can optimize LSPR characteristics for specific applications.^22−24^ These insights underscore the potential of chiral plasmonics not only to deepen our understanding of light–matter interactions but also to inspire innovative technologies across multiple disciplines.^25−27^

In the realm of chiral nanostructures, three primary categories can be distinguished. First, there are intricate three-dimensional (3D) structures, such as helices, which inherently exhibit strong chirality regardless of the observer’s perspective (intrinsic chirality). Second, it is also possible to observe chiral responses in periodic arrays of nonchiral motifs at certain angles of incidence (extrinsic chirality).^28,29^ Finally, there are two-dimensional (2D) chiral elements such as gammadia, spirals, L-shapes, etc., characterized by a symmetry plane along the out-of-plane *z*-axis. The symmetry is typically disrupted by the presence of a substrate or structural imperfections,^30^ resulting in a chiral response when measured under normal incidence. This latter group exhibits a strong interaction with light, leading to various intriguing effects like broken time reversal^31^ or asymmetric light transmission.^32^ However, the complexity of the fabrication processes employing time-consuming and costly methods, such as electron beam lithography (EBL)^4^ or focused ion beam (FIB), has limited the exploitation of these architectures in actual devices.^33^ Soft lithography-based techniques are emerging as a viable alternative for fabricating chiral plasmonic structures, offering the advantages of reduced production costs and increased throughput. Prior studies have demonstrated the potential of these methods, including nanoparticle self-assembly for creating chiral structures^34^ and the combination of nanoimprinting or nanosphere lithography with angled deposition to achieve chiral response.^35,36^

This work introduces a new set of chiral metasurfaces consisting of 2D arrays of inverted pyramids whose bases are intentionally rotated with respect to the lattice vectors of the array. The “misalignment” between the base of the pyramid with respect to the 2D lattice results in a strong chiral response of the metasurface. These metasurfaces are produced by combining soft lithography with the established silicon chemical etching technique. The resulting 2D chiral geometry engraved in the silicon wafer can be easily replicated in poly(dimethylsiloxane) (PDMS) stamps and thus seamlessly replicated in polymer films. This simple and cost-effective fabrication process does not involve expensive equipment, clean room facilities, or time-consuming procedures. The chiral metasurfaces are imprinted onto thin resist films and subsequently metal-coated to achieve a robust chiral response within the visible spectrum. Complete polarimetric characterization using the Mueller matrix method reveals CD values surpassing 23,500 mdeg, which are among the highest values reported for plasmonic nanostructures.^37^ Furthermore, the metasurfaces exhibit exceptional Kuhn dissymmetry factors (g-factors) approaching unity and circular birefringence above 350°/μm. Numerical simulations using the finite-difference time-domain (FDTD) method support the optical measurements and provide insights into the effect of geometric parameters on the chiro-optical activity of the structures.

## Experimental and Computational Methods

**Figure 1:**
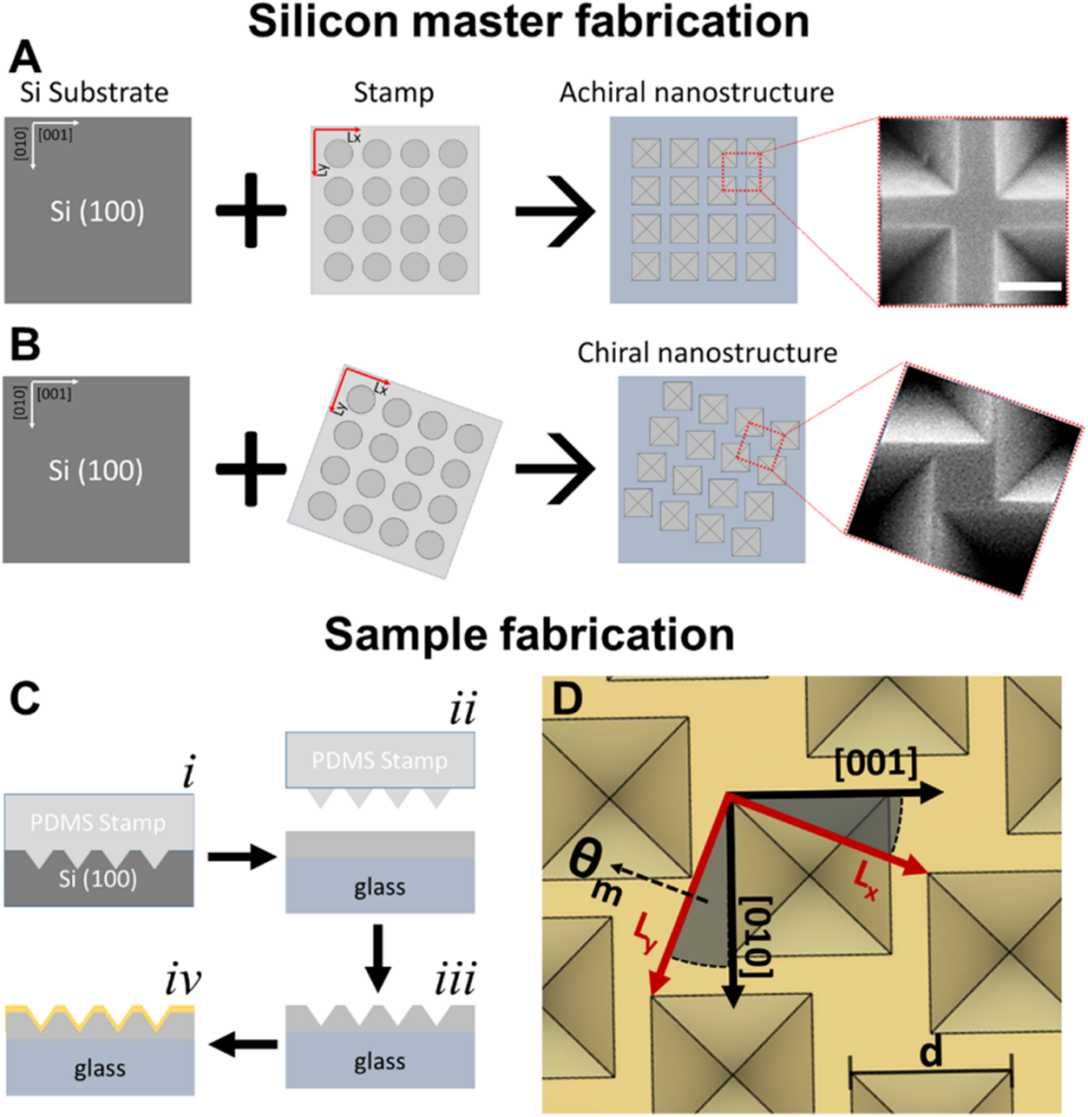
Fabrication of chiral arrays of nanoscale inverted pyramids. Step 1: Silicon master fabrication. Scheme and scanning electron microscopy (SEM) images for (A) θ_m_ = 0° (achiral) and (B) θ_m_ ≠ 0° (chiral) architectures. Step 2: (C) Fabrication of plasmonic chiral metasurfaces on glass substrates. (i) Transfer of nanostructures from silicon masters to PDMS stamps, (ii) and (iii) NIL of SU8 on glass, and (iv) thermal evaporation of a metallic layer. (D) Geometrical parameters of the architecture: Mismatch angle (θ_m_) defined as the angle between Si crystallographic directions and array lattice vectors (**L**_**x**_ and **L**_**y**_). The lattice parameter (**L**_**x**_, **L**_**y**_) is 600 nm, and the pyramid lateral size is d. Scale bar in part (A) is 200 nm.

This study encompasses the design, fabrication, characterization, and electrodynamic modeling of chiral metasurfaces. The fabrication process involves two main steps: (1) the creation of the chiral array on a silicon (100) wafer using soft lithography and chemical etching and (2) the replication of the chiral patterns onto elastomeric molds acting as printing stamps for polymeric films. Further details of each step are provided in the Supporting Information (SI). In short, the original chiral array on silicon starts with the creation of a square array pattern (directional vectors **Lx** and **Ly**) of cylindrical holes in a SU8 photoresist layer on a Ge-coated silicon substrate (Figures 1A,B and S1). The 2D pattern on the substrate is made by nanoimprint lithography (NIL) and using a prepatterned PDMS mold. The 150 nm Ge layer serves as a protective mask for Silicon in a subsequent wet etching step.^38^ Next, a reactive ion etching (RIE) step transfers the pattern from the SU8 resist to the silicon underneath, etching both the polymer and the thin Ge layer in the holes of the pattern. The exposed silicon undergoes anisotropic chemical etching,^39^ resulting in inverted square pyramids engraved on the silicon wafer due to the differing etching rates of different crystallographic planes (Figure S1). The pyramid base sides consistently align with the **[100]** and **[010]** crystallographic directions regardless of pyramid size or etching conditions.^40^ Once the chiral masters are obtained, PDMS molds replicating the corrugated surface are produced and will be used as printing stamps on nanostructure polymer films (Figure 1C). In our case, the chiral geometries are replicated onto a SU8 photoresist thin film deposited on glass. Finally, the chiral plasmonic array is obtained by metal coating the films via thermal evaporation (details and atomic force microscopy (AFM) characterization are in Figure S2).

The originality of our work lies in the way in which chirality is introduced into the square array of inverted pyramids. During the nanostructuration of the silicon wafer (step 1), the lattice vectors (**L**_**x**_ and **L**_**y**_) of the square array of holes are rotated with respect to the crystallographic directions of the silicon wafer by a mismatch angle (θ_m_), as shown in Figure 1D. When θ_m_ = 0°, the resulting unit cell appears like a cross with *C*_4_ symmetry (a 4-fold rotational axis) (Figure 1A, SEM image). However, for mismatch angles θ_m_ between 0 and 45°, the *C*_4_ symmetry is maintained (Figure 1B SEM image), but the structure resembles a chiral motif without mirror symmetry, giving rise to chiro-optical properties.

**Figure 2:**
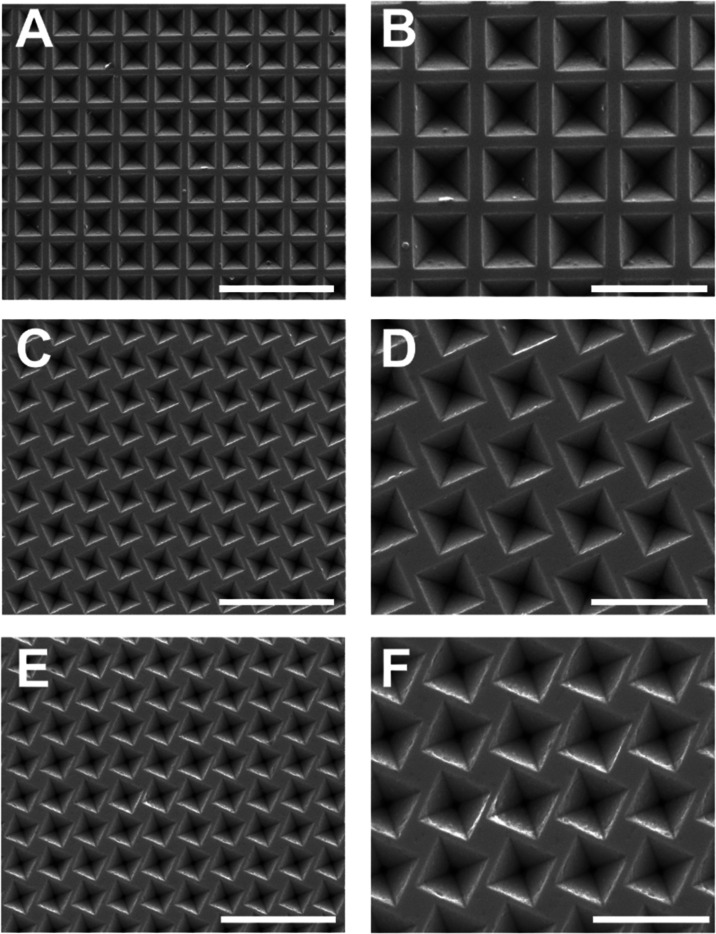
SEM images of inverted pyramid arrays at various magnifications etched in a Silicon (100) substrate (A, B) with a mismatch angle (**θ**_**m**_) of 0°, (C, D), of −23°, and (E, F) of +24°. The lattice parameter of the array is 600 nm. Scale bars in panels (A, C, E) and (B, D, F) are 2 and 1 μm, respectively.

Figure 2 shows the SEM images of silicon masters patterned with achiral and chiral inverted pyramid arrays (additional SEM images are available in Figure S3). The top row (Figure 2A,B) depicts structures fabricated with a mismatch angle (θ_m_) of 0°. The middle row (Figure 2C,D) and bottom row (Figure 2E,F) show chiral structures created with mismatch angles θ_m_ of −23 and +24°, respectively. This process leads to an outstanding uniformity in the fabricated structures, as proven by the statistical analysis. The relative percentage standard deviation of pyramid size (*d*) is below 3%. The average lateral size of the pyramids is 460, 441, and 461 nm for each row, respectively. The corresponding standard deviations are 7.9, 11.9, and 11.6 nm. Since the pyramid depth is directly related to its lateral size (*h* = (1/√2)*d*), *h* is expected to exhibit a similar low deviation. These results demonstrate that the combined approach of NIL followed by anisotropic chemical etching of silicon coated with a Ge hard mask is a powerful method for high-throughput nanofabrication of metasurfaces with chiral motifs.

**Figure 3:**
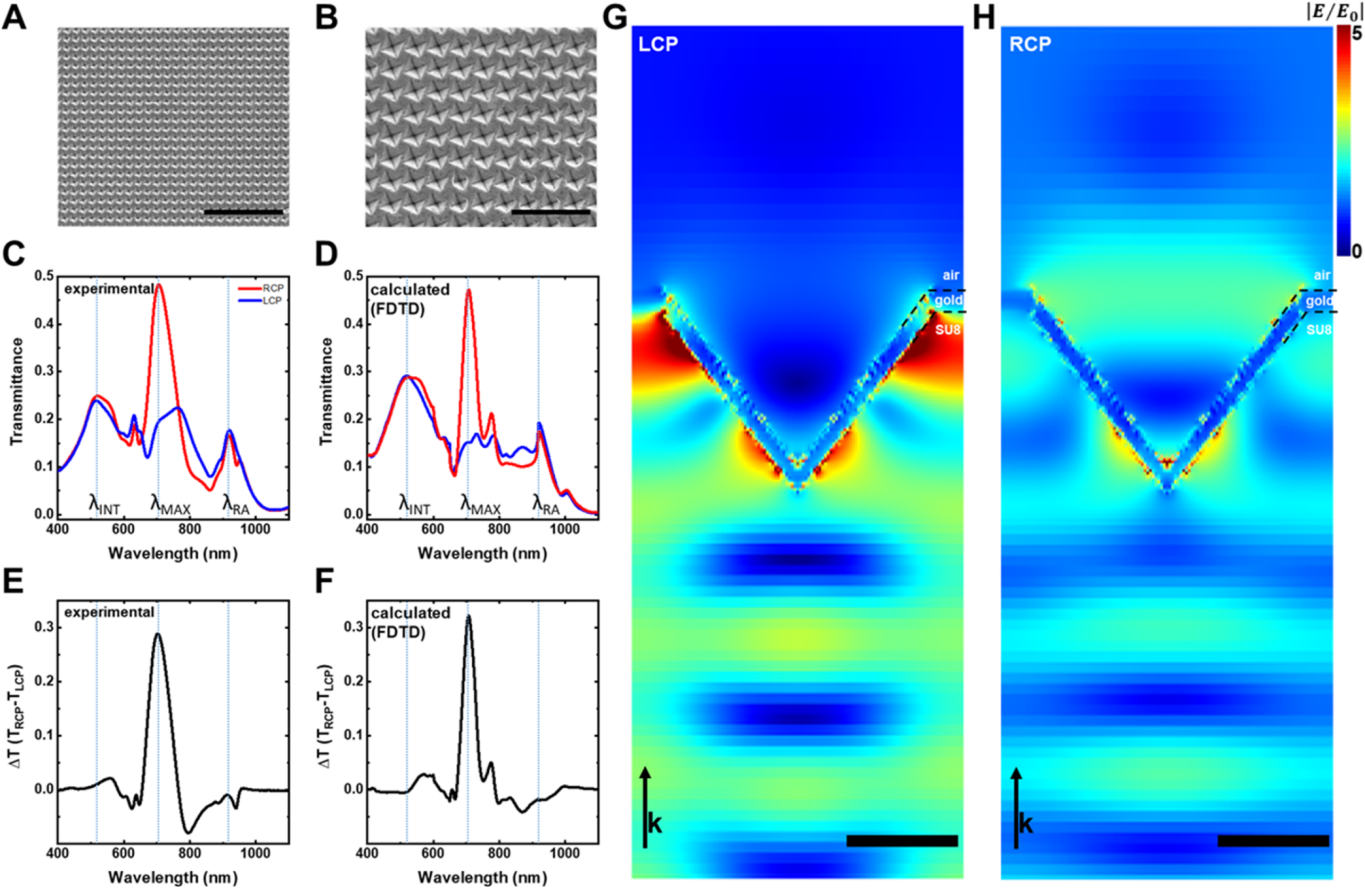
SEM images and optical properties of plasmonic chiral metasurfaces. (A, B) SEM images of gold-coated patterned SU8 on top of a glass substrate. Lattice parameter of 600 nm and pyramid size of 461 ± 12 nm. Scale bars in panels (A, B) are 5 and 2 μm, respectively. (C) Experimental and (D) calculated transmittance spectra. Left circular polarized (LCP) and right circular polarized (RCP) incident light spectra are shown by blue and red lines, respectively. (E, F) experimental and calculated differential transmittance (Δ*T* = *T*_LCP_ – *T*_RCP_) extracted from panels (C, D). Vertical dashed lines at (C–F): λ_INT_ = 521 nm, λ_MAX_ = 705 nm, and λ_RA_ = 930 nm. FDTD calculated electric field distribution cross section (*xz*) at λ_MAX_, for (G) LCP and (H) RCP. FDTD model structure parameters LP: 600 nm, pyramid size: 450 nm, mismatch angle: 24°, Au thickness: 40 nm, substrate *n* = 1.53, λ = 710 nm. Scale bars in panels (G, H) are 200 nm.

The pyramid arrays in silicon are later used as masters, from which elastomeric molds featuring the negative pattern are made. Briefly, the silicon wafer is functionalized with an antistick layer, and then PDMS (Sylgard 185) is poured and cured on top. These PDMS molds are later used to corrugate 500 nm thin films of SU8 (Microchem 2000.5) by hot embossing.^35^ The corrugated SU8 films are later covered with gold via thermal evaporation (see Figure 3A,B).

The optical properties of gold-coated samples were investigated by using visible–near-infrared (vis–NIR) spectroscopy. By measuring the transmittance of these samples under circularly polarized light, we determined their chiral characteristics. A custom-built optical setup was used to illuminate the samples with either left- or right-circularly polarized light. For a more comprehensive polarimetric analysis, a 4-photoelastic modulator (PEM) Mueller matrix polarimeter was employed in transmission mode. To validate our experimental results, we performed numerical simulations using the finite-difference time-domain (FDTD) method (Ansys FDTD-Solutions, Lumerical). These simulations considered both forward and backward propagation of light through the samples under normal incidence conditions.

## Results and Discussion

### Optical Properties of the Inverted Pyramid Arrays

The experimental and calculated optical properties of gold-coated inverted pyramid arrays depicting chiral motifs (θ_m_ = 24°) are presented in Figure 3. Panels C and D correspond to the experimental and calculated transmittance spectra, whereas the computed CD, obtained from the differential transmittances, is displayed in panels E and F. The FDTD simulated electromagnetic near-field distribution across the structure is depicted for both circular polarizations at 705 nm in panels G and H; Figure 3C presents the experimental transmittance spectra for LCP light (blue line) and RCP light (red line) over the spectral range of 400–1100 nm. The experimental spectra show remarkable agreement with the FDTD numerical simulations (Figure 3D), with only minor deviations attributable to inevitable sample imperfections. The observed transmittance values are significantly high, reaching up to 50% for the RCP component, beyond the transmittance of a flat 40 nm thick Au coating taken as a reference (see Figure S4). The different transmittance values found for both light handednesses underscore the pronounced chiral response of the chiral metasurface.

The optical response of the pyramid arrays shows three clear features that we indicated with vertical dashed lines in Figure 3C–F. The first maximum in transmittance is observed for both LCP and RCP at **λ**_**INT**_ = 521 nm, corresponding to the spectral region just above the Au interband transitions.^41^ The maximum transmittance for **RCP** light is observed at **λ**_**MAX**_ = 705 nm, revealing a differential coupling between the incident RCP and LCP light in the metamaterial structure. Finally, the **λ**_**RA**_ = 930 nm corresponds to the first order of the lattice Rayleigh-Wood anomaly, an optical phenomenon that indicates the onset of the diffraction orders in a grating. These anomalies manifest at specific wavelengths where the diffracted light propagates parallel to the array’s surface, resembling a guided wave. The first-order Rayleigh anomaly (λ_RA_) plays a crucial role in determining the spectral region in which diffraction occurs. In square lattices, this threshold wavelength, denoted as λ_RA_, is given by the formula
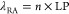
where *n* is the refractive index of the substrate and LP is the lattice parameter of the array. Essentially, λ_RA_ marks the boundary between the diffractive (λ ≤ λ_RA_) and nondiffractive spectral regions (λ > λ_RA_).^42^

Across the wavelength range of 520–930 nm, the CPL handedness significantly impacts the spectral profiles, as evident from both theoretical and experimental spectra (Figure 3C,D). The most pronounced difference in transmittance between the two circular polarizations occurs around **λ**_**MAX**_. To quantify this difference, the spectral dependence of Δ*T* (Δ*T* = *T*_RCP_ – *T*_LCP_) is illustrated in Figure 3E,F. The FDTD simulations predict an unusual Δ*T* exceeding 30% (Figure 3F), which aligns well with experimental observations and is further supported by the FDTD electric field distribution cross sections at **λ**_**MAX**_ shown in Figure 3G,H. This achievement underscores the strong chiro-optical response of the inverted pyramid square lattice. Light propagating through the substrate interacts differently with the nanostructured surface depending on its polarization. For LCP light, the electric field is mostly confined within the substrate (Figure 3G), leading to a lower transmittance. Conversely, RCP light depicts a stronger electric field in the transmitted region (Figure 3H), resulting in higher transmittance. (see Figure S5 for electric field distribution at different cross sections). It is worth noting that the remarkably high Δ*T* values found in these lossy metallic films are notable, since these kinds of plasmonic structures typically show low transmittance (Figure S4).

Another feature of the spectra observed in Figure 3C,D is that the transmittance difference for both polarizations is minimal at the edges of the displayed wavelength range. For wavelengths shorter than **λ**_**INT**_, the structured array exhibits a transmittance similar to a flat Au layer (Figure S4). This can be primarily attributed to the dominant Au interband transitions in this spectral range, which diminishes the relevance of the morphological effects.^43^ This becomes evident in the observed Δ*T*, which progressively approaches zero as the wavelength decreases. Similarly, the Δ*T* reduces beyond λ_RA_. The Rayleigh-Wood anomaly of the lattice is determined by the lattice parameter multiplied by the effective refractive index of the substrate (SU8 over glass). In sum, the diffraction seems to strongly influence the chiral activity of the samples (for further discussion, see Figure S15).

### Chiral Properties Dependence on the Mismatch Angle (θ_m_)

**Figure 4:**
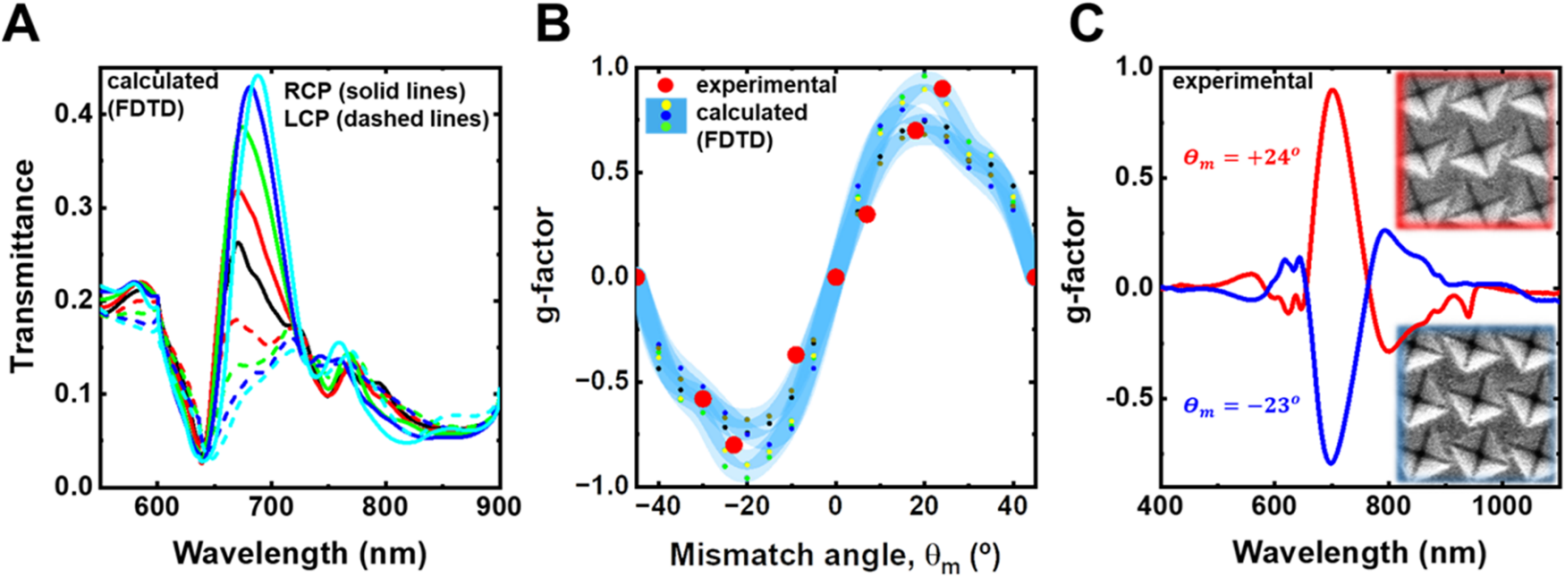
(A) Calculated transmittance spectra at normal incidence of plasmonic chiral metasurfaces. Left circular polarized (LCP) (dashed lines) and right circular polarized (RCP) (solid lines), the color of the lines corresponds to the mismatch angle **θ**_**m**_ = 0° (black), 5° (red), 10° (green), 15° (blue), 20° (cyan). FDTD parameters LP: 600 nm, pyramid size: 450 nm, **θ**_**m**_: 22°, Au thickness: 50 nm, substrate *n* = 1.53. (B) calculated g-factor dependence on mismatch angle (shaded blue curve with colored dots) (for *d* = 450 (green), 475 (yellow), 500 (black), 510 (gray), and 525 nm (blue)) and experimental g-factor values obtained in samples with different **θ**_**m**_ (red dots). (C) Enantiomorph behavior: g-factor spectra for **θ**_**m**_ = +24° (red line) and **θ**_**m**_ = −23° (blue line). Inset: SEM images of each Au-coated sample.

The mismatch angle between the bases of the pyramids and the main directions of the lattice clearly governs the chiral behavior of the nanostructures presented in this manuscript. Those mismatch angles between 0° and ±45° result in arrays with different chiroptical responses, as supported by a parametric study using FDTD. The resulting transmittance spectra for both polarizations are displayed in Figure 4A. For clarity, only results between 0 and 20° are plotted. Continuous lines correspond to the spectral response under RCP light, whereas dashed lines represent those under LCP. The transmittance shows a strong dependence on the θ_m_, with the most pronounced modulation observed between 650 and 720 nm.

As expected for a symmetric structure (θ_m_ = 0°, black lines), the transmittance spectra are identical for both right- and left-circularly polarized light, exhibiting a maximum transmittance of 26% at λ = 670 nm. Breaking this mirror symmetry with a small tilt angle (θ_m_ = 5°, red lines) introduces a differential interaction with right-handed circularly polarized light. This is evident from the emergence of a differential transmittance of 14%, while the wavelength of maximum transmittance remains around 671 nm. Further increasing the mismatch angle (θ_m_ = 10, 15, and 20°) induces a continuous increase in Δ*T*, reaching 25, 31, and 33%, respectively. Furthermore, a gradual red shift of the maximum transmittance is observed, shifting from λ = 670 nm at θ_m_ = 0° to λ = 687 nm at θ_m_ = 20° (see Figure S6A for the complete range of θ_m_).

To identify the optimal mismatch angle θ_m_, the Kuhn dissymmetry factor (*g*-factor) was calculated at the wavelength of maximum transmittance for each nanostructure. The *g*-factor is a widely used metric for comparing chiro-optical response, defined as
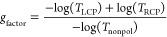
where *T*_RCP_ and *T*_LCP_ represent the transmittance for right-circularly polarized and left-circularly polarized light, respectively, and *T*_nonpol_ is the transmittance for nonpolarized light.^44^

Figure 4B (shaded blue line) presents the calculated g-factor values for different pyramid sizes (ranging from 450 to 525 nm, colored dots) across the range of mismatch angles. The graph highlights that a positive θ_m_ is the enantiomorph (mirror image) of the one obtained with its negative θ_m_. The g-factor reaches a local maximum between 20 and 25°. This finding is consistent with recent observations by De Leon et al., where they achieved a Δ*T* exceeding 2% with a maximum chirality at 22.5° in a metasurface design.^23^ Their design employed a square array of unit cells with four opposing rods, where the maximum response occurred between nanostructure scattering and array diffraction directions. Gryb et al. also explored a similar architecture composed of an array of rotated a-Si rectangles, achieving a maximum chiral response at comparable angles.^45^

The maximum dissymmetry factor values in this study fall slightly below unity (*g*-factor ≈1). To put this value in perspective, typical values for molecular systems are around 1 × 10^–3^,^46−48^ while macromolecules exhibit values around 0.01.^49^ In the field of plasmonic nanostructures operating in the visible region, g-factor values exceeding 0.3–0.5 are fairly uncommon.^20,21,50−53^ The red data dots in Figure 4B represent the experimental *g*-factors obtained in the fabricated structures (see Figure S6B for the complete set of *g*-factor spectra), in excellent agreement with the FDTD simulated values. Structures with tilt angles (θ_m_) close to ±22.5° exhibit the highest g-factors, reaching values close to −0.8 and +0.9, respectively. A more extensive analysis of the geometrical parameters (*d*, LP, metal film thickness for gold or silver) is presented in Section 8 of the SI (Figures S7–S9).

Figure 4C shows the g-factors obtained for two enantiomorphic metasurfaces fabricated with tilt angles of θ_m_ = +24° (red line) and −23° (blue line). The inset provides SEM images of these structures. Remarkably, despite the minimal mismatch angle difference, the response is clearly mirrored across the wavelength range 400–900 nm, as expected for enantiomers. The g-factor profiles exhibit similar magnitudes but opposite signs, even for the fine spectral features observed near 650 and 800 nm. Only minor discrepancies are found around the Rayleigh-Wood anomaly wavelengths. This exceptional agreement among the enantiomeric responses highlights the robustness of the presented metasurface fabrication method. To the best of our knowledge, this represents one of the highest-performing chiral systems where such a distinct enantiomorphic response is achieved.

### Influence of Mismatch Angle on Near-Field Optical Properties

**Figure 5:**
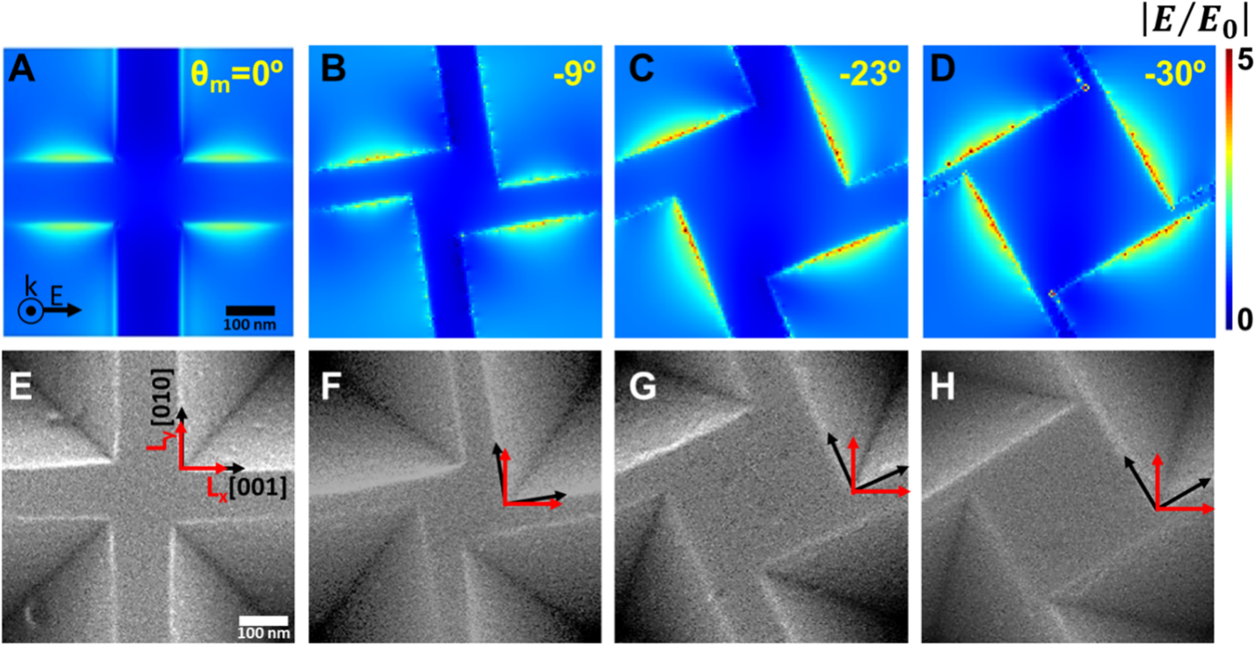
(A–D) Calculated near-field distribution at the upper plane of the metasurfaces with different **θ**_**m**_, 0, −9, −23, and −30°. LP = 600 nm, and Au thickness = 50 nm. The pyramid size is *d* = 470, 515, 456, and 495 nm from panels (A–D), respectively. The incident light is linearly polarized with the electric field vector in the **L**_**x**_ direction. (E–H) SEM images of the nanostructures modeled in panels (A–D);^54^ the **Lx** and **Ly** vectors are shown in each panel with the corresponding Si crystalline directions (**[001]**, **[010]**). Scale bar: 100 nm.

In order to gain a deeper understanding of the underlying mechanisms responsible for generating chirality, we performed FDTD simulations for structures originating from various mismatch angles (0, −9, −23, and −30°). The geometrical parameters of the modeled nanostructures were retrieved from SEM characterization.^54^Figure 5 presents the modeled near-field distributions alongside the corresponding SEM images. The calculations assumed linearly polarized light propagated through the substrate with an electric field vector in the **L**_**x**_ direction. The resulting electric field distributions near the nanostructures exhibit a strong dependence on the θ_m_.

As shown in Figure 5A, an achiral structure, illuminated with linear polarized (achiral) light,^44^ produces a symmetrical near-field distribution with respect to the **L**_**y**_ direction. In contrast, for nonzero mismatch angles (θ_m_ ≠ 0) shown in Figure 5B–D, the electric field distributions not only increase in magnitude but also acquire characteristics of chirality as θ_m_ increases. Notably, the field distribution in Figure 5C (θ_m_ = −23°) exhibits a preferential handedness resembling *C*_4_-symmetry, similar to the nanostructure itself and the plasmonic mode excited by circularly polarized light (see Figure S5).

In order to quantify the qualitative observations, a correlation was established with the calculated normalized optical chirality factor (**C/C**_**0**_) of the light after interaction with the metasurface. The optical chirality factor is a quantity that reflects the handedness of light, particularly in its interaction with chiral materials. This magnitude is defined as
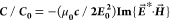
where μ_0_ is the vacuum magnetic permeability, ***c*** is the speed of light in vacuum, E⃗^*^ represents the complex conjugate of the electric field, and ***H⃗*** is the magnetic field strength. The optical chirality (*C*) is normalized by *C*_0_, where *C*_0_ is the optical chirality of the incident RCP light.^55^ The upper limits for *C*/*C*_0_ in the far field are −1 and +1 for perfect LCP and RCP light, respectively. However, in a lossy medium, the maximum achievable value is proportionally reduced by the factor (*T*/*T*_0_), where *T*/*T*_0_ represents the ratio of transmitted and incident light intensity.

The *C*/*C*_0_ factors were calculated for the inverted pyramid arrays in the far-field transmitted light. In particular, for θ_m_ = 0, *C*/*C*_0_ equals 0, while for nanostructures with θ_m_ ≠ 0, the *C*/*C*_0_ values become more negative as θ_m_ increases. When θ_m_ = −23°, the maximum value is *C*/*C*_0_ = −0.116. It is noteworthy that *C*/*C*_0_ for the incident linearly polarized light is zero. Thus, the degree of chirality acquired by the light is induced by its interaction with chiral nanostructures.^56^ In Figure S10, the values *C*/*C*_0_ obtained for −45° ≥ θ_m_ ≥ 45° are presented; the trend closely resembles the one observed for the *g*-factor values (Figure 4C), with the maximum close to ±22.5°.

### Intrinsic Chirality in Inverted Pyramid Arrays

**Figure 6:**
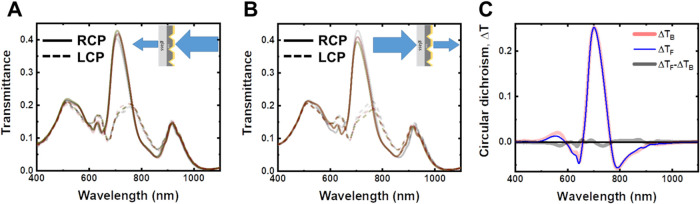
(A, B) Experimental transmittance spectra at normal incidence of the plasmonic chiral metasurface at different positions within the sample under RCP (solid lines) and LCP (dashed lines) light. The illumination propagation is shown in the inset. (A) From the air/metal interface and (B) from the air/glass interface. (C) Δ*T* spectra for each propagation direction blue line (forward) and pink line (backward). The gray line represents the Δ*T*_F_ – Δ*T*_B_.

With the aim to ascertain the intrinsic nature of the observed chirality in the inverted pyramid metamaterial, transmittance measurements were performed with light impinging from both directions (air and glass sides). The measurements taken from different spots in the sample to illustrate homogeneity are shown in Figure 6A from the metallic layer (forward direction) and Figure 6B from the substrate (backward direction) for LCP (gray lines) and RCP (red lines) light (see *insets schemes*). The resulting circular dichroism spectrum represented by Δ*T* and presented in Figure 6C for both propagation directions (red, forward, Δ*T*_F_ and pink, backward, Δ*T*_B_) displays minimal discrepancies, as indicated by the black line oscillating around zero (Δ*T*_F_ – Δ*T*_B_). The invariance in spectral behavior is indicative of the intrinsic chirality achieved in these architectures and is observed in all samples, including those with varying geometric parameters (Figure S11). Furthermore, measurements performed at various azimuthal angles of the sample, as observed in Figure S12, reveal negligible differences between the spectra. This underscores the absence of linear in-plane artifacts in the spectral response.

### Complete Polarimetric Characterization

**Figure 7:**
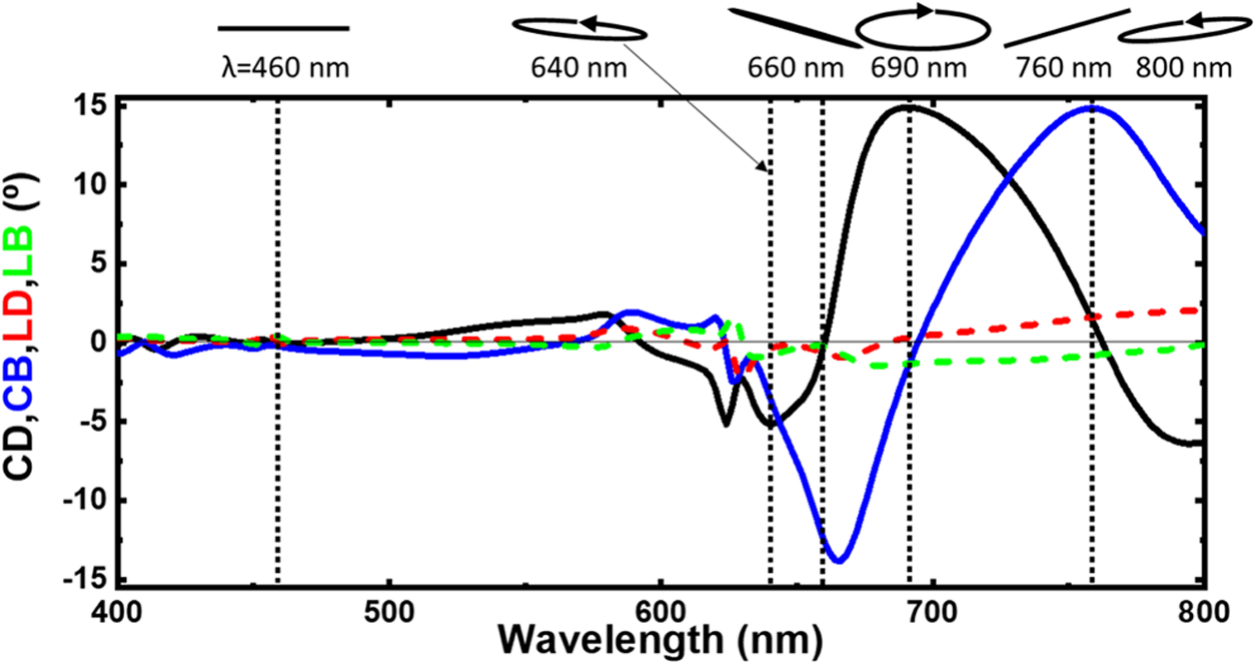
Polarimetric characterization of inverted pyramid metasurfaces with θ_m_ = 24°. Circular dichroism (CD, black line), circular birefringence (CB, blue line), linear dichroism (LD, red dashed line), linear birefringence (LB, green dashed line), calculated from the Mueller Matrix elements, and polarization ellipses calculated from the Mueller Matrix elements for 6 wavelengths, considering the incidence of linearly polarized light parallel to the *x*-axis. LP = 600 nm, d = 450 nm, and Au layer = 40 nm.

A comprehensive polarimetric analysis of the system was undertaken by acquiring the Mueller matrix in normal transmission. The Mueller matrix method is a tool used to describe how light interacts with a material. It captures the complete picture by considering not just the intensity but also the polarization state of light before and after interacting with the sample. From the mathematical point of view, the (4 × 4) Mueller matrix is a transfer matrix between two (4 × 1) Stokes vectors representing the initial and final polarization states. This allows for understanding how a sample/structure affects the polarization of light, revealing valuable information such as the CD and ORD.^21^ The Mueller matrix elements obtained for a pair of enantiomorphs are illustrated in Figure S13. The optical activity exhibited by these architectures fundamentally relies on the symmetries of specific Mueller matrix elements, M_03_ = M_30_ and M_12_ = −M_21_. Given that the remaining off-diagonal elements responsible for linear components are substantially weaker, M_03_, M_30_ and M_12_, and M_21_ account for circular dichroism (CD) and circular birefringence (CB), respectively. Details about the experimental determination of the Mueller matrix elements are provided in the SI. Figure 7 presents the observed CD (black line) and CB (blue line), alongside linear dichroism (LD) (red dashed line) and linear birefringence (LB) (green dashed line). It has to be noted that the linear components are negligible compared to the circular ones, which is mainly due to the cell architecture design with *C*_4_-symmetry in a square lattice. While this condition poses a challenge for many reported structures, the inverted pyramid array offers highly circular-dependent properties with minimal linear effects, making it a promising solution. It is also worth noting that Mueller matrix characterization of the inverted pyramid metasurfaces indicates very low depolarization of the samples (Figure S14).

The CD and CB values are notably high within the 600–800 nm range, and the characteristic *Cotton effect* is clearly observed: when the dichroism is at its maximum, the birefringence is approximately zero and vice versa. Such behavior is clearly evident in the polarization ellipses plotted for different wavelengths (Figure 7 top panel) when illuminated with horizontal linearly polarized light. Particularly, at λ = 690 nm, CD reaches its maximum values while the birefringence is close to zero, resulting in transmitted light exhibiting elliptical polarization, with its major axis in the horizontal plane. Conversely, at λ = 660 and 760 nm, the birefringence reaches its maximum values and the dichroism approaches zero; only ORD is observed, with rotation values exceeding ±15°. An ORD of 15° is a remarkable degree of rotation per unit length of 375°/μm. This achievement is particularly noteworthy, not only in comparison to natural materials but also relative to nanostructures possessing similar characteristics. Finally, at λ = 460 nm, the transmitted light preserves its incident polarization (linear in the horizontal plane) owing to the proximity of CD and CB to zero. At other wavelengths, the effects are milder and blended, resulting in rotated ellipses (i.e., λ = 640 and 800 nm).

### Benchmarking

**Figure 8:**
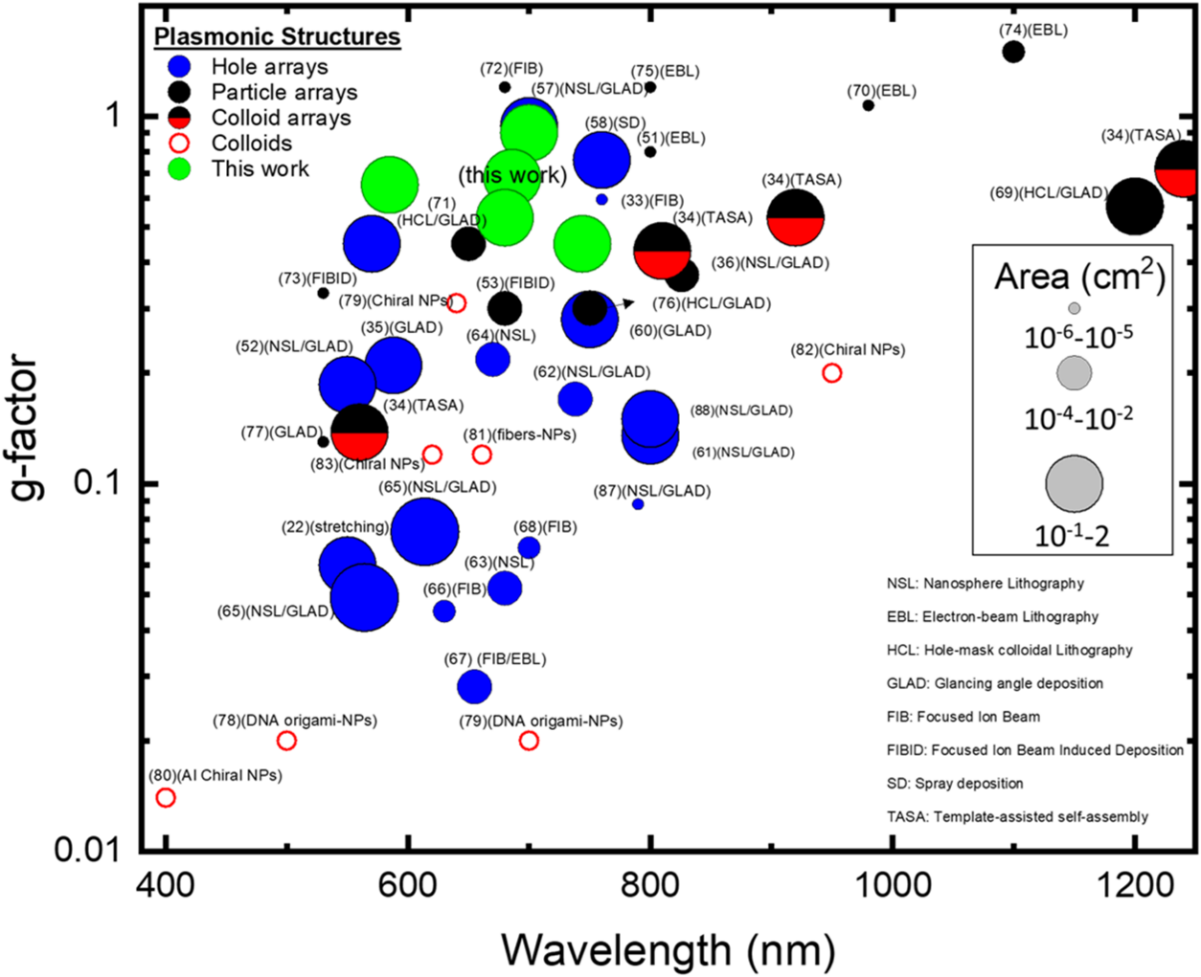
Performance comparison as a function of wavelength and sample patterned area for chiral plasmonic structures fabricated using different fabrication techniques, including plasmonic films (represented by blue circles, where the size of the circle represents the samples area), plasmonic nanoparticle arrays (red circles), and plasmonic colloids (red empty circles).^22,33,35,36,51−53,57−83,87,88^

In this final part, we place our work within the broader context of the existing chiral plasmonic structures. To this purpose, a graph illustrating the g-factor as a function of wavelength has been constructed (Figure 8). This graph incorporates data from previously reported structures, with a particular focus on those composed of plasmonic films (represented by blue circles, where the size of the circle represents the sample area),^22,33,35,52,57−68^ as well as plasmonic nanoparticle arrays (red circles)^36,51,53,69−77^ and colloids (red empty circles)^78−83^ for a comprehensive comparison. The fabrication technique used for each structure is also indicated. Notably, the presented structures surpass most plasmonic film counterparts, achieving g-factors approaching unity at 700 nm and exceeding 0.6 across various parameter combinations within the 600–720 nm range. This exceptional performance is further highlighted by contrasting it with the limitations of existing approaches. Our approach offers distinct advantages: high g-factors, broad operational wavelength range, large-scale fabrication compatibility (≈1 cm^2^ samples), superior cost-effectiveness and scalability, and high versatility for diverse applications. Metal nanoparticle arrays, while achieving high g-factors, often operate in the NIR with restricted sample areas due to complex fabrication methods.^4,74,84−86^ Plasmonic colloids require specific synthesis conditions and often need substrate support, potentially negatively impacting their chiral performance. Our simple two-step fabrication method (imprinting and metal deposition) overcomes these limitations, making the inverted pyramid arrays a promising new class of high-performance, scalable, and cost-effective chiral metamaterials.

## Conclusions

We have demonstrated the outstanding chiroptical performance of twisted arrays of inverted pyramid metasurfaces fabricated via a high-throughput protocol integrating nanoimprint lithography and silicon anisotropic chemical etching. The metasurfaces, crafted on resins and covered with metallic deposits, exhibited a consistently uniform response across substantial square centimeter areas, showcasing both efficiency and scalability in their production. The predominant chiral properties were found to be primarily dictated by the angle formed between the lattice vectors of the array and the orientation of the square base of the pyramid. The designed architectures manifest 3D behavior marked by intrinsic chirality, mediated through the preferential diffraction of light in relation to their handedness. Optimization of key parameters in the metasurface architecture led to remarkable chiroptical performances, with g-factors nearing unity, an optical rotation dispersion (ORD) of 375°/μm, and circular dichroism values exceeding 23°. These results place the chiral responses among the highest observed for plasmonic film-type structures, demonstrating the potential for significant advancements in the field. The scalability of our fabrication method sets it apart from existing techniques, offering a cost-effective and highly reproducible pathway for producing large-area, high-performance chiral metasurfaces. Beyond fundamental insights, this work holds broad implications for the development of next-generation optical and nanophotonic devices. Furthermore, the integration of these structures into devices such as polarimeters, optical filters, and light modulators could offer new capabilities in both research and industry. Challenges in achieving higher precision in the fabrication process or fine-tuning the chirality in certain configurations may require further optimization. Additionally, exploring the tunability of the optical response across a broader range of wavelengths and different materials to further enhance the chiroptical properties is an essential next step.

## Supporting Information Available

Details on materials and fabrication procedure; additional SEM images; FDTD calculations; a comprehensive study on the chiral inverted pyramids arrays geometrical parameters optimization; full Mueller matrix elements; optical characterization; and discussions about the chirality mechanisms in metasurfaces (PDF)
