# Supplementary material for: Strong Chiro-Optical Activity of Plasmonic Metasurfaces with Inverted Pyramid Arrays
Source: ACS Appl Mater Interfaces. 2025 Mar 3;17(10):15824–35. doi: 10.1021/acsami.4c19803 (PMC11912205; doi:10.1021/acsami.4c19803)
Supplement: Supplementary file 1 — am4c19803_si_001.pdf [file am4c19803_si_001.pdf]

Supporting Information

Strong Chiro-Optical Activity of Plasmonic Metasurfaces with Inverted Pyramid Arrays

Luis Alberto Pérez[1]*, Jinhui Hu[1], Jose Mendoza-Carreño[1], Miquel Garriga[1], Maria Isabel Alonso[1], Oriol Arteaga[2], Alejandro R. Goñi[1,3]* and Agustín Mihi[1]*

1: Institute of Materials Science of Barcelona, ICMAB-CSIC, Campus de la UAB, 08193 Bellaterra, Catalonia, Spain

2: Department of Applied Physics, PLAT group, University of Barcelona, 08028 Barcelona, Spain

3: ICREA, Passeig Lluís Companys 23, 08010 Barcelona, Spain

Corresponding authors: lperez@icmab.es, goni@icmab.es, amihi@icmab.es

Index

- 1. Materials 2
- 2. Fabrication of the Chiral Inverted Pyramid Arrays on Silicon Substrates 2
- 3. Fabrication of the Plasmonic Chiral Inverted Pyramid Arrays on SU8/glass Substrates 4
- 4. Additional SEM Images 5
- 5. Optical Properties Measurements 7
- 6. Numerical Simulations – FDTD 7
- 7. ΔT and g-factor Dependence on the Mismatch Angle θ_m 9
- 8. Chiral Inverted Pyramid Arrays Geometrical Parameters Optimization 10
- 9. C/C0 Dependence on the Mismatch Angle θ_m 16
- 10. Additional Information: Temporal Reversibility and Complete Polarimetric Characterization 17
- 11. Chirality Mechanisms in Inverted Pyramid Arrays Metasurfaces 20
- 12. Angular Resolved Transmittance Characterization 27
- References 29

1. Materials

Prepatterned silicon masters with cylindrical holes were purchased from CEMITEC (Spain). Hard polydimethylsiloxane (PDMS) silicone elastomer kits were purchased from Gelest (Morrisville, PA) and a soft PDMS Sylgard184 silicone elastomer kit from Dow Corning Corporation (Auburn, MI). 1H,1H,2H,2H-Perfluorooctyltrichlorosilane (PFOTS, 97%) was purchased from Alfa Aesar (Thermo Fisher GmbH, Karlsruhe, Germany). Glass substrates were purchased from LABBOX, Spain. SU8 2000.5 was purchased from Microchem. Gold and silver pellets were purchased from Kurt. J. Lesker.

2. Fabrication of the Chiral Inverted Pyramid Arrays on Silicon Substrates.

2.1. Preparation of Original Master (Cylindrical Holes)

Silicon substrates (masters) patterned with hole arrays (area: 1 cm^2) were used as purchased from CEMITEC (Spain). Lattice parameters were 500 and 600 nm, hole diameter 298 and 338 nm, respectively. The depth in both patterns was 390 nm. The masters were silanized with an anti-sticking layer by PFOTS (2 μL in the desiccator for 20 min) to prevent the adhesion of photoresists and ensuring a easy release during replication.

2.2. Preparation of Cylindrical Pillar Arrays Molds

PDMS molds (a negative replica of the original master) were fabricated following a previously reported protocol.[1] The molds were fabricated by casting a thin layer of hard-PDMS on the master followed by a thick layer of soft PDMS as support, PDMS was cured at 110 °C for 2 h.

2.3. Fabrication of Inverted Pyramids Arrays

Arrays of inverted pyramids on Si substrates were fabricated combining nanoimprint lithography (NIL) steps with Si anisotropic chemical etching steps, following the fabrication protocol developed previously reported.[2] As it can be observed in the scheme in figure S1, the key fabrication steps include: *i*) A 150 nm thick Ge layer MBE deposition onto a polished silicon (100) wafer, as a hard mask.[3] *ii*) On top of the Ge layer, a 600 nm thick SU8 photoresist layer was spin-coated at 2000 rpm for 10 s. *iii*) PDMS stamps fabricated according to section 2.2, were used to imprint a square array of holes into the SU8 photoresist: As presented in the manuscript figure 1A, B, different angles between the silicon crystalline directions and the square arrays directions (mismatch angle, $\boldsymbol{\theta_m}$) from -30º to 30º were used in order to fabricate architectures with chiral properties, this was attempted by changing the azimuthal angle of the PDMS stamps respect to the Silicon substrate crystal orientation. The PDMS mold was gently placed on top of the substrate with the SU8 layer at 90 °C and after 10 sec left to cool down to below the glass transition temperature of the resist and then the PDMS mold was removed. The patterned SU8 layer was cured under UV light for 10 min and hard-baked at 160 °C on a hot plate for 30 min. *iv*) Reactive ion etching (RIE) using a PlasmaPro Cobra 100 system (Oxford Instruments) was employed to remove the resist residual layer and the Ge hard mask inside the holes. Two recipes were used: to remove the residual resist (O_2 plasma: 50 sccm, 12 mTorr and HF power: 50 W for 2 min) and the

Ge layer (O_2/SF_6: 5/30 sccm, 15 mTorr and HF power: 75 W for 2 min). *v*) Silicon anisotropic wet etching using a 33 wt% KOH solution was used to get the inverted pyramid arrays. The etching was performed at 85 °C during 30 seconds, followed by a quickly rinse with fresh water. *vi*) Finally, the residual Ge mask was then removed using 30% H_2O_2 for 20 min, rinsed with water and dry blown with N_2.

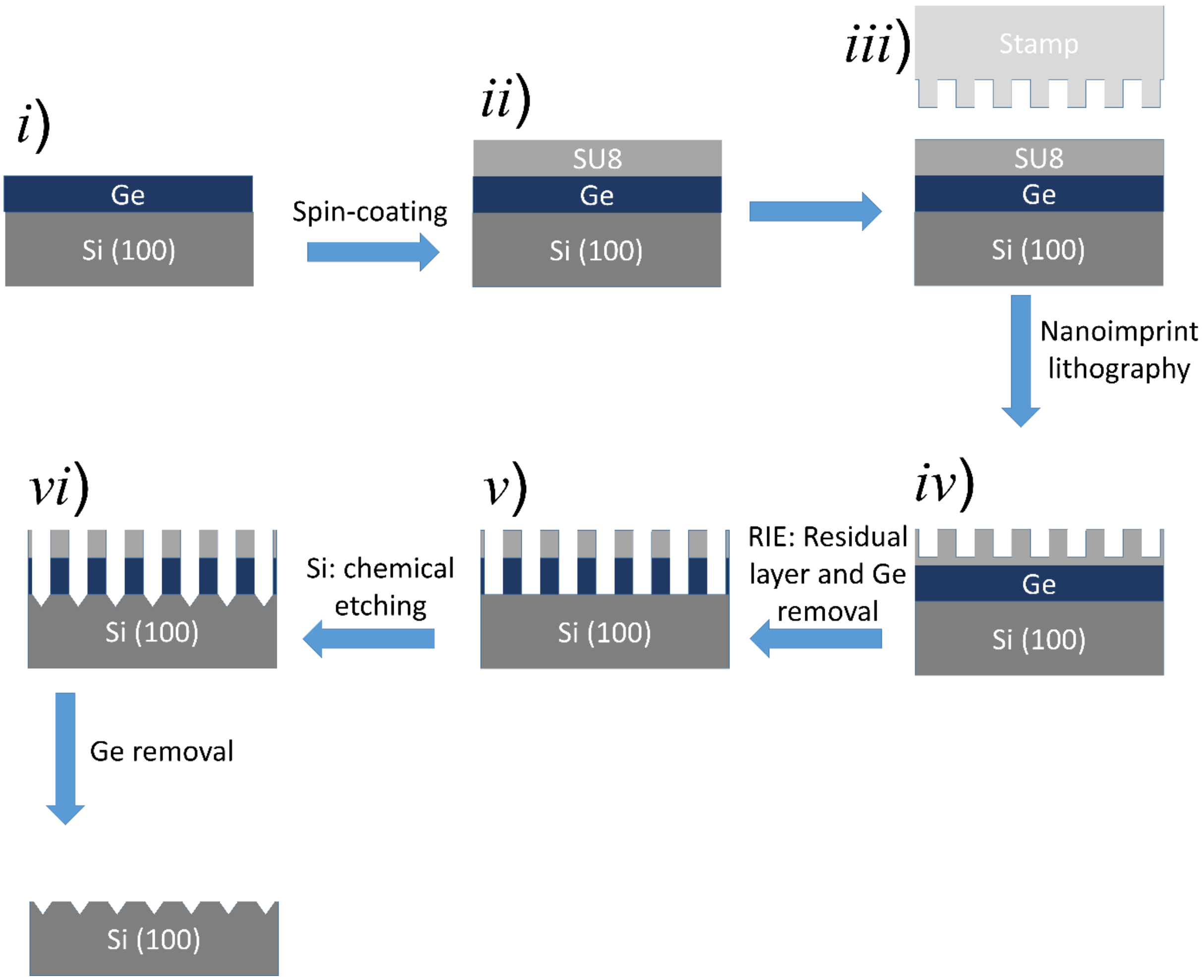

Figure S1 *Fabrication of the chiral inverted pyramids architecture on silicon substrates. i) Ge layer deposition using MBE, ii) SU8 layer spin coating, iii) NIL patterning of the SU8 layer, iv) RIE residual layer removal, v) SI anisotropic chemical etching vi) Ge residual layer removal.*

3. Fabrication of the Plasmonic Chiral Inverted Pyramid Arrays on SU8/glass substrates

The patterned Si substrates with the ordered array of inverted pyramids with different mismatch angles, serve as primary masters for the fabrication of the plasmonic metasurfaces. Multiple replicas of the master can be produced, without compromising the quality of the inscribed architecture. This allowed us the simultaneous production of the various plasmonic metasurfaces.

3.1. Preparation of Silicon Master and Up-right Pyramids PDMS Molds (Figure S1 step i)

Silicon substrates obtained as described in section 2.3, were silanized with an anti-sticking layer using the procedure described in section 2.1, followed by the replication of the inverted pyramid arrays using PDMS molds as described in section 2.2, obtaining PDMS stamps with ordered arrays of up-right pyramids.

3.2. Patterning of the SU8/glass Substrates and Metal Deposition. (Figure S1 steps ii-iv)

The SU8 photoresist layer was spin-coated on top of a clean glass at 2000 rpm for 10 s, the obtained thickness was 600 nm. The PDMS stamps produced following section 2.2 procedure, were used to imprint a square array of inverted pyramids into the SU8 photoresist. The PDMS mold was gently placed on top of the substrate with the SU8 layer at 90 °C and after 10 sec left to cool down to below the glass transition temperature of the resist and then the PDMS mold was removed. The patterned SU8 layer was cured under UV light for 10 min and hard-baked at 160 °C on a hot plate for 30 min.

Finally, a 30 - 90 nm thick metal layer was deposited on the surface of the inverted pyramids by thermal evaporation. The pressure in the evaporation chamber was kept below 1×10^{-6} mbar, the deposition rate for Au was 0.06 nm/s. Ag layers were deposited at 5×10^{-7} mbar and 1 nm/s.

3.3. Cross Section and Top View Scheme of the Chiral Inverted Pyramid Arrays

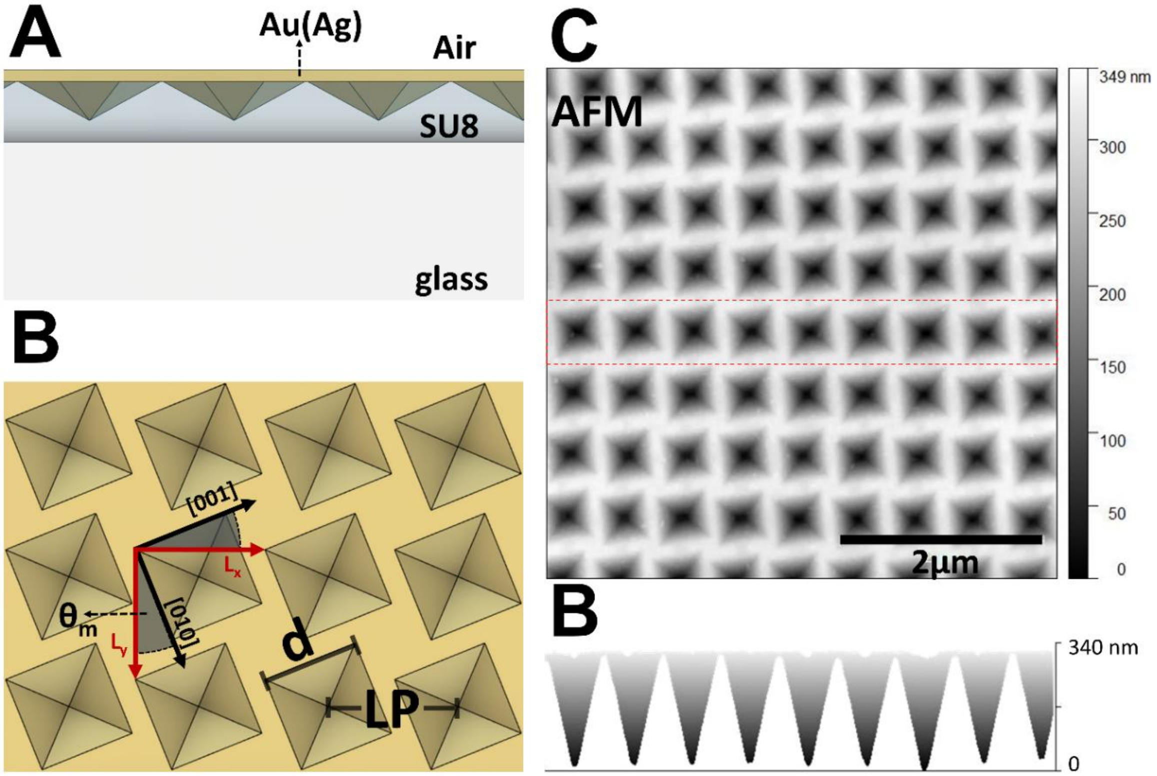

Figure S2 *(A) Cross section and (B) top view schematic representations of the chiral metasurfaces. (A) A glass substrate is coated with a patterned SU8 layer. A metallic layer is deposited onto the SU8 patterned layer. (B) Geometrical parameters of the chiral inverted pyramid arrays: lattice parameter (LP), size of the pyramids base (d) and the mismatch angle (θ_m). The θ_m is defined as the angle between the silicon crystalline directions [001] and [010], and the array unit vector directions (Lx, Ly). (C) AFM image of a sample with* $\boldsymbol{\theta_m}$ *= -9º covered with 40 nm gold layer. (D) Cross-sectional view extracted from the AFM image, as indicated by the dashed red rectangle.*

4. Additional SEM Images

Scanning electron microscopy (SEM) images were collected by FEI QUANTA 200 Field Emission Gun microscope, operating at an acceleration voltage of 15 kV. The working distance was 10 mm. All images were obtained in high vacuum and detecting the secondary electron signal. In figure S3 a series of SEM images of the silicon masters and the samples can be observed. The magnifications 10000x, 20000x, 50000x and 100000x, are displayed as four. Masters and samples with different mismatch angles are shown in: (A-D) $\boldsymbol{\theta_m}$ = -30º, (E-L) $\boldsymbol{\theta_m}$ = -23º (M-P) $\boldsymbol{\theta_m}$ = -9º, (Q-T) $\boldsymbol{\theta_m}$ = 0º, (U-X) $\boldsymbol{\theta_m}$ = 7º, (Y-AB) $\boldsymbol{\theta_m}$ = 18º, (AC-AJ) $\boldsymbol{\theta_m}$ = 24º. Panels (E-H) and (AG-AJ) correspond to images of SU8/glass substrates coated with 40nm of Au, the NIL in these samples was performed using the PDMS molds corresponding to the masters shown in (I-L) and (AC-AF), respectively. It can be observed that the topography of the surface is perfectly transferred from the Si substrate to the SU8 layer.

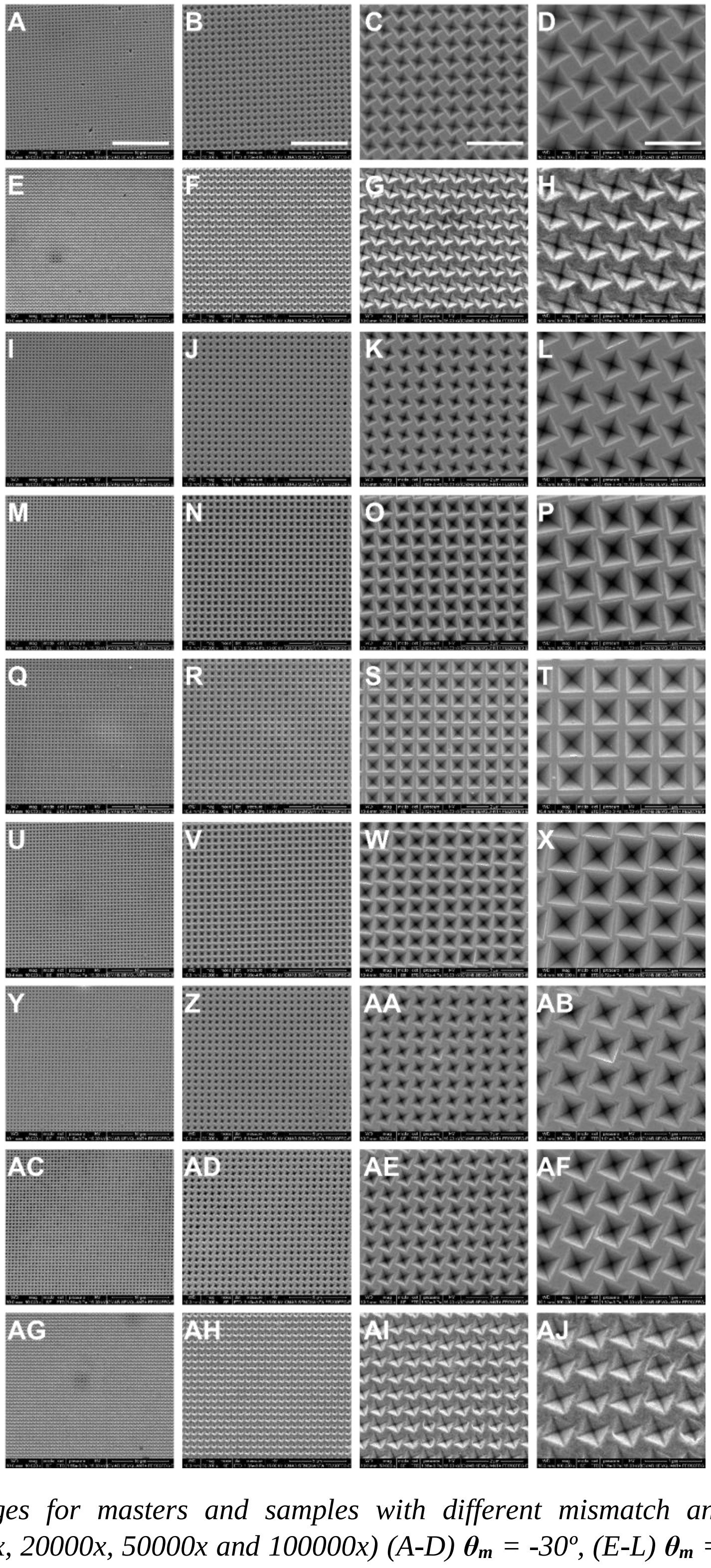

Figure S3 *SEM images for masters and samples with different mismatch angle (*$\boldsymbol{\theta_m}$*) at different magnifications (10000x, 20000x, 50000x and 100000x) (A-D)* $\boldsymbol{\theta_m}$ *= -30º, (E-L)* $\boldsymbol{\theta_m}$ *= -23º (M-P)* $\boldsymbol{\theta_m}$ *= -9º, (Q-T)* $\boldsymbol{\theta_m}$ *= 0º, (U-X)* $\boldsymbol{\theta_m}$ *= 7º, (Y-AB)* $\boldsymbol{\theta_m}$ *= 18º, (AC-AJ)* $\boldsymbol{\theta_m}$ *= 24º. (E-H) and (AG-AJ) correspond to SU8/glass substrates coated with 40nm of Au, corresponding to the same geometrical parameters to (I-L) and (AC-AF) respectively. Lattice parameter: 600 nm The scale bars for the columns starting in A, B, C, D are 10, 5, 2 and 1 µm, respectively.*

5. Optical Properties Measurements

5.1. Transmittance and Circular Dichroism Measurements

Transmittance measurements were obtained in a home-built optical setup. A tungsten halogen lamp (Ocean Optics, HL-2000-HP) was used as light source, the unpolarized light passes through a Glan-Thompson linear polarizer, followed by a superachromatic quarter-wave plate (Thorlabs, SAQWP05M-700, 325 - 1100 nm) with the fast axis oriented at $\pm\pi/4$ or 0 with respect to the vertical direction, generating left- and right- (L-/R-) circularly polarized (CP) or linearly polarized light. The quarter waveplate was placed onto rotation mounts (Thorlabs ELL14). Light was focused to the sample using a 10x objective (NA = 0.1). The transmitted light was collected by a fiber coupled spectrophotometer (Ocean Insight, QEPro-FL (400 – 1100 nm). The CD was calculated as the transmittance difference between right and left circular polarized light, $\Delta T = T_{RCP} - T_{LCP}$ and the dissymmetry factor (g-factor) as:

$$g_{factor} = 2\left(\left(-log(T_{LCP}) + log(T_{RCP})\right)/\left(-log(T_{LCP}) - \log(T_{RCP})\right)\right). \quad (1)$$

6. Numerical Simulations - FDTD

All simulations were performed with the Finite Difference Time Domain (FDTD) commercial software (Ansys FDTD-Solutions, Lumerical). Numerical simulations were performed at normal incidence with the light propagating from the glass substrate side (backward direction) or from the air side (forward direction). Linearly polarized light was modelled as a single plane wave source with its corresponding electric field vector direction. For circularly polarized light modeling, two sources with the electric field vector aligned to the x- and y-axis, respectively, and a phase shift of $\pm\pi/2$ to generate LCP or RCP, were used. The wavelength range was 400 - 1100 nm. A substrate with a single inverted pyramid was set as the unit cell for the simulations, with the values of geometrical parameters (d, LP, θ_m) extracted from the experimental microscopy images characterizations. Periodic boundary conditions were imposed at the grating plane and perfect-matched layer in the normal axis.

6.1. Flat and Structured Surfaces

The unpolarized light transmittance spectra, calculated for flat Au films and arrays of chiral inverted pyramid metasurfaces, are depicted in Figure S4. The spectra corresponding to flat layers are represented by dashed lines, while those of nanostructured surfaces are shown with solid lines. Three thicknesses of the metallic film are presented: 70 nm (black lines), 50 nm (blue lines), and 30 nm (red lines). In contrast to the flat layers, the spectra of the metasurfaces exhibit a complex structure within the depicted range, with significantly higher transmittances observed for wavelengths beyond 510 nm. At shorter wavelengths, the interband transitions of Au result in a diminished influence of the geometric factor, rendering the transmittance of the structured surfaces comparable to that of the flat layers.[4]

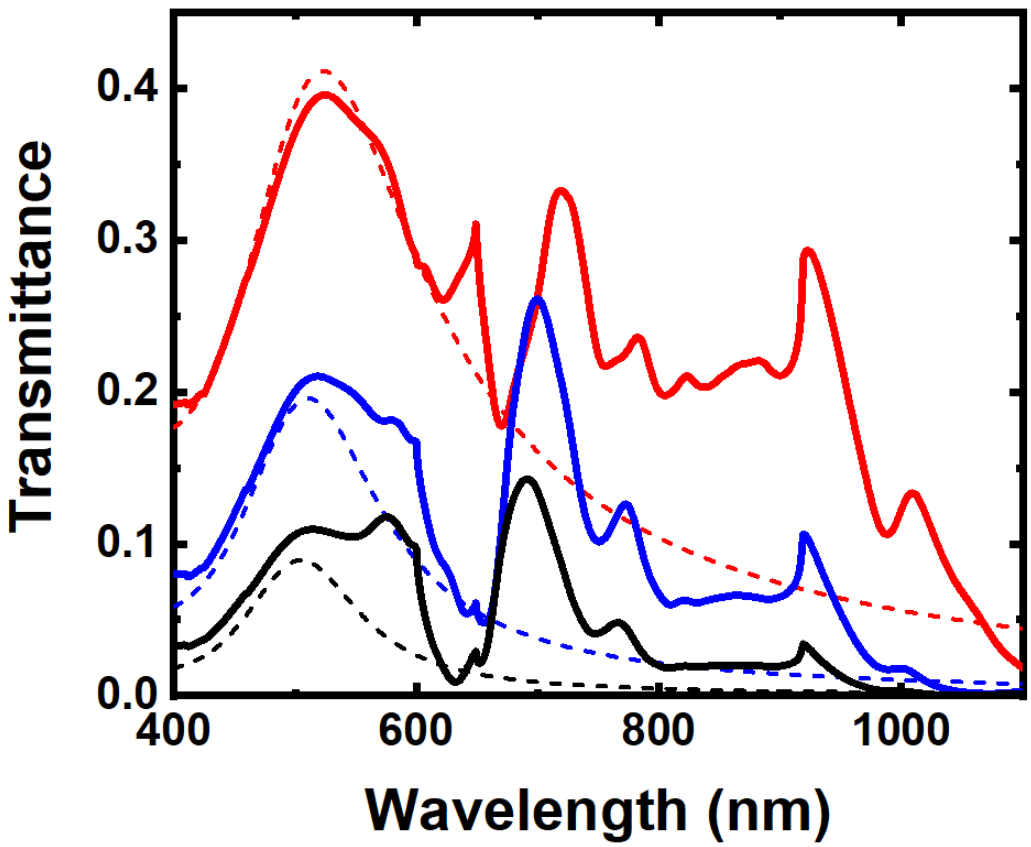

Figure S4 *Transmittance spectra for non-polarized light for flat Au films (dashed lines) and chiral inverted pyramid arrays (solid lines). The thickness of the metal layer is 70, 50 and 30 nm corresponding to black, blue and red lines, respectively. The pyramids size is 450 nm, LP = 600 nm and* $\theta_m = 22º$.

6.2. Electric Field Cross Section Distributions at Different Wavelengths

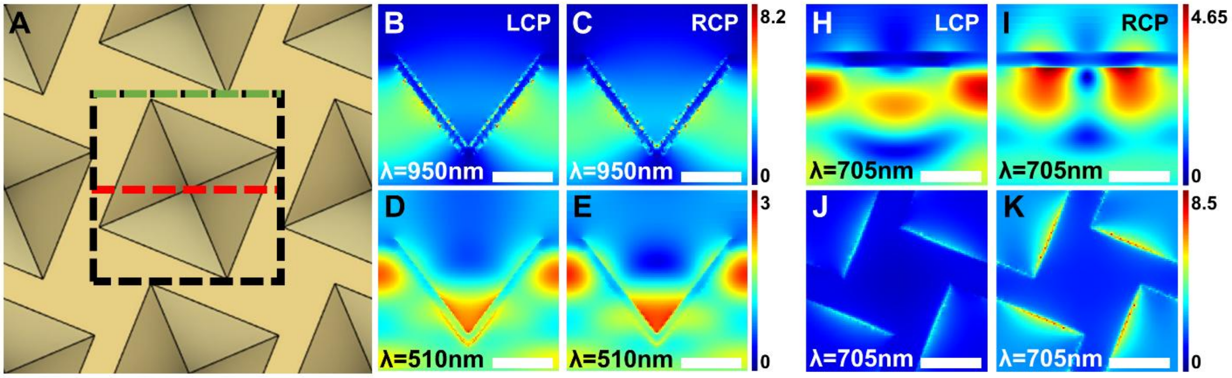

Figure S5 *FDTD calculated electric field distribution cross section in a chiral inverted pyramids array under LCP and RCP light. FDTD model structure parameters LP: 600nm, pyramid size: 450 nm, mismatch angle: 22º, Au thickness: 40nm, substrate n=1.53. Top view scheme of the inverted pyramids array, the dashed line and square, represent the regions where* $\vec{E}$ *is calculated: (B-E) and (figure 3G, H) red line, xz cross section, (H, I) green line, xz cross section, and (J, K) black square, xy cross section. The wavelength in B, C is 950 nm, in D, E 510 nm, and in H-K) 705 nm. Scale bars in (B-K) are 200 nm.*

7. ΔT and g-factor Dependence on the Mismatch Angle

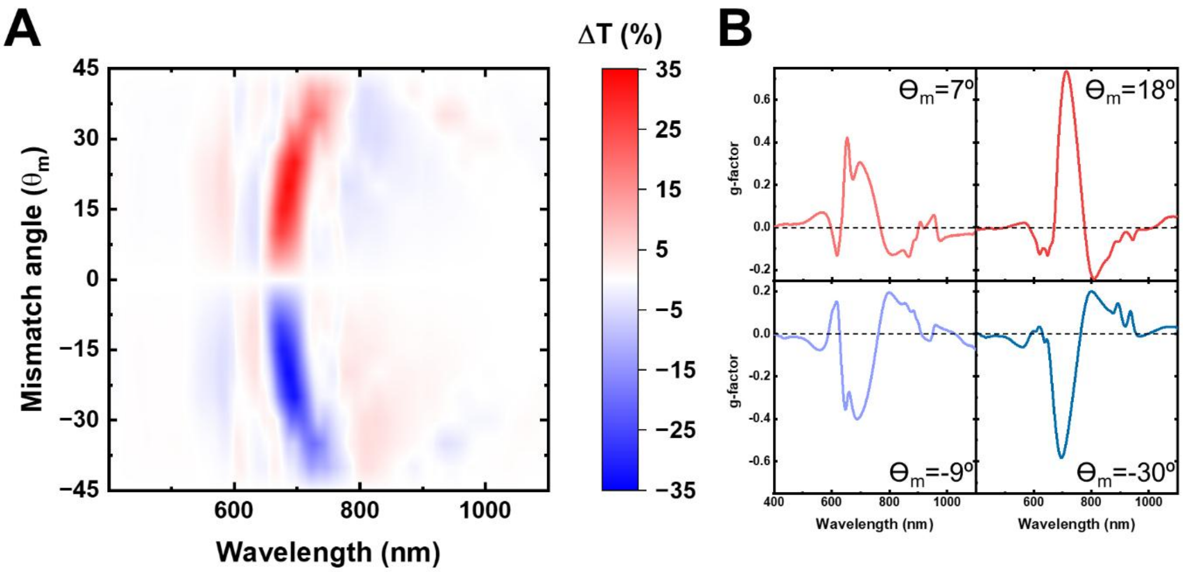

Figure S6 *(A) FDTD differential transmittance spectra* $\Delta T = T_{RCP} - T_{LCP}$ *vs. the mismatch angle* θ_m *for a chiral inverted pyramid arrays. FDTD structure parameters LP: 600nm, pyramid size: 450 nm, Au thickness: 50nm, substrate n=1.53. (B) Experimental g-factors spectra for chiral inverted pyramids arrays with various* θ_m*.*

8. Chiral Inverted Pyramid Arrays Geometrical Parameters Optimization

8.1. Influence of Pyramid Dimensions *(d)*

The size of the inverted pyramids within the metasurface architecture is a critical geometric parameter. These pyramids not only support plasmonic and photonic modes, but also dictate the three-dimensional configuration of the metasurface. To avaluate their impact on chiral properties, a systematic computational study was conducted. The lateral dimension (d) of the pyramids was varied systematically, while all other parameters remained constant. The resulting transmittance for LCP and RCP light, along with ΔT and g-factor maps as a function of pyramid size, are presented in Figure S7.

The calculations revealed a strong influence of pyramid size on the chiral characteristics of the metasurfaces. Notably, for a lattice parameter of 600 nm and a mismatch angle of -22°, the g-factor (and ΔT) remained relatively low for pyramids with d < 200 nm (Figure S7D-F). However, beyond d = 250 nm, the g-factor exhibited a significant increase, reaching a maximum around d = 450 nm. Interestingly, further increases in d led to a decline in the g-factor, likely due to increasing interactions between neighboring pyramids and their incipient overlap.

This behavior deviates from the common trend observed in many optical properties of lattices, where optimal unit cell element sizes tend to be smaller or mid-range relative to the lattice parameter, as exemplified by the Q-factor.[5] In the case of chiral activity and the g-factor specifically, larger lattice elements approaching the lattice parameter appear to be more favorable. To shed light on this phenomenon, a geometric analysis was performed. Figure S7C schematically depicts the "metamorphosis" of the metasurface morphology with increasing pyramid size. While small pyramids (100-200 nm) have minimal impact on the overall array properties, the interstitial regions between pyramids in the 300-450 nm size range progressively resemble "fused gammadions" (Figure S7C) This intriguing structural transformation coincides with the observed enhancement in the chiral response. However, in the case of larger pyramids, they overlap, leading to the loss of this distinct feature. Incidentally, producing these sizes could exceed the limitations of the chemical fabrication process. From the experimental point of view, too large pyramid sizes would cause potential issues with uncontrolled etching, resulting in overlap during fabrication.

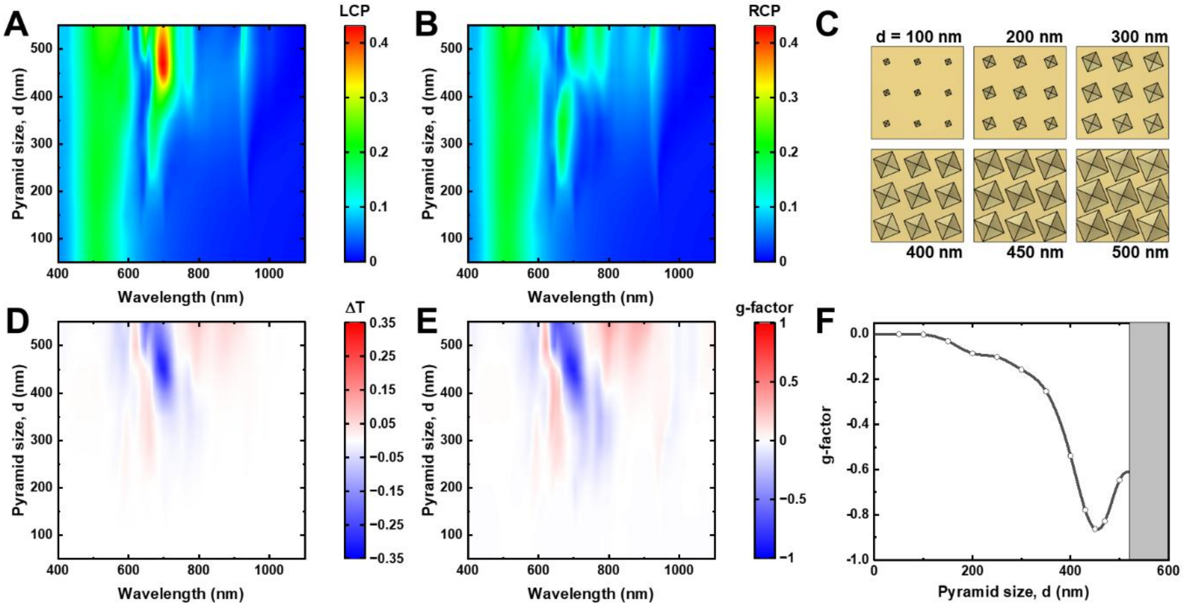

Figure S7 *Influence of the pyramid size on metasurfaces properties using the following FDTD model parameters LP: 600nm, mismatch angle: -22º, Au thickness: 50nm, substrate refractive index n=1.53. (A, B) Calculated transmittance spectra maps as a function of pyramid size (d), for LCP (A) and RCP light (B). (C) top view schematic of metasurfaces with increasing pyramid sizes (from 100 to 500 nm). (D, E) ΔT and g-factor maps as a function of pyramid size (d). (F) Dependence of the maximum g-factor on the size of the pyramids (d).*

8.2. Influence of Metal Film Thickness on the Chiral Response

As previously observed in Figure S4, the transmittance intensity is dependent on the thickness of the metal film. Structured surfaces exhibit enhanced transmittance compared to their flat counterparts. The chiral response of the metasurfaces is also significantly influenced by the metal thickness.

Figure S8A presents experimental transmittance spectra for LCP and RCP light incident on metasurfaces with a lattice parameter of 600 nm, pyramid size of 441 nm, and mismatch angle of -23°. The experimentally measured thicknesses of the metallic films for each pair of spectra: (i) 79.8 nm, (ii) 63.5 nm, (iii) 40.6 nm, and (iv) 27.5 nm.

A clear trend emerges: decreasing the thickness of the metallic film leads to an increase in the transmittance for both LCP and RCP light. The magnitude of |ΔT| exhibits a corresponding increase as the film thickness decreases from 79.8 nm to 40.6 nm, rising from 8% to 27.6%. Additionally, a redshift of the wavelength corresponding to the maximum |ΔT| is observed (see Table S1). However, further reduction in the metal film thickness results in a pronounced decrease in |ΔT|.

The trend observed for $|\Delta T|$ is consistent with the observed for the |g-factor|. The optimal thickness range lies between 40-50 nm, yielding values close to 0.9 and 0.8 for calculations and experiments, respectively (Figure S8B).

<u>*Alternative Metric for Chiral Characterization: ΔT/T*</u>

In addition to the g-factor, a complementary metric frequently employed for the characterization of chirality in thin-film solid systems is ΔT/T.[6] This parameter normalizes the observed magnitude of ΔT by the overall (non-polarized) transmittance (T), resulting in a dimensionless value ranging from 0 to 2. This normalization facilitates another convenient comparison of the degree of circular dichroism across samples exhibiting varying levels of overall light transmission

For illustrative purposes, the metasurfaces possessing a metallic layer thickness of 63.5 nm, can be analyzed. As depicted in Figure S8C, this sample exhibits a maximum ΔT of 15.8% at a wavelength (λ) of 684 nm. The corresponding transmittance values for RCP and LCP light are 6.7% and 22.5%, respectively. When evaluated using the ΔT/T metric, this sample yields a value of 1.07. Interestingly, the highest recorded ΔT/T value within this sample is 1.35, observed at λ = 667 nm. At this wavelength, the transmittance values are T_{RCP} = 2.9% and T_{LCP} = 15%. These values translate to a circular dichroism signal of CD = 23.5° (23500 mdeg), indicating a strong enrichment of one circular component (84% LCP) in the transmitted light. However, it is noteworthy that the g-factor for this specific wavelength is not the maximum (g-factor = 0.6) (Table S1)

These observations highlight the existence of key distinctions in the trends exhibited by the g-factor and ΔT/T. The differences in the values become particularly significant when analyzing systems with either low transmittance (indicative of high extinction) or exceptionally high transmittance (indicative of low extinction).

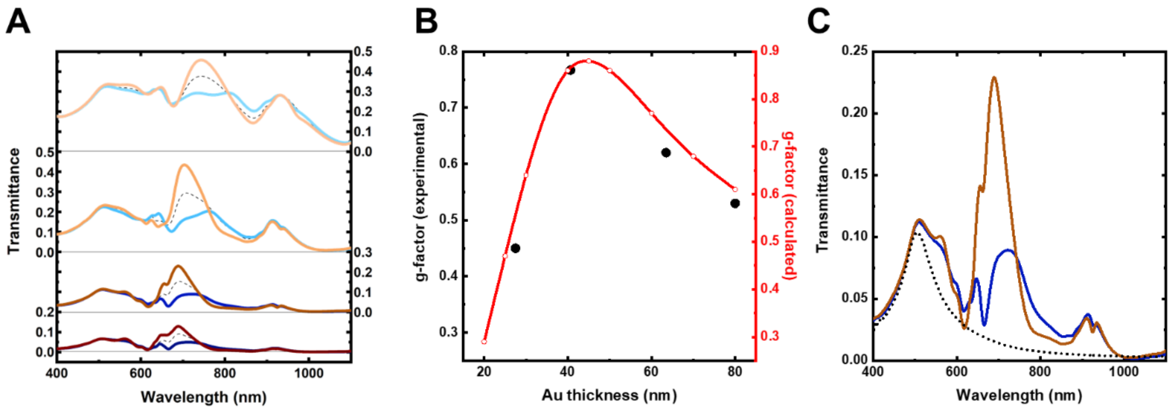

Figure S8 *Influence metal layer thickness on metasurface properties. (A) Experimental transmittance spectra for LCP (red to orange), and RCP light (blue to sky-blue) at normal incidence on metasurfaces with varying Au layer thicknesses: i) 79.8, ii) 63.5, iii) 40.6, and iv) 27.5 nm. LP= 600 nm, d= 441 nm,* θ_m *= -23°. (B) g-factor dependence on the Au layer thickness. The red line represents calculated values, and the black dots represent experimental values calculated from the spectra in panel (A). (C) Experimental transmittance spectra at normal incidence of a plasmonic chiral metasurface with a LP = 600 nm, coated with 63.5 nm of Au. The solid lines show the transmittance for LCP (orange) and RCP (blue). The black dotted lines represent the transmittance of a flat region outside the patterned area (for reference).*

Table S1 *Optical parameters obtained from samples with different metal layer thickness. LP = 600 nm, d = 441 nm,* θ_m *= -23°. The maximum transmittance* T_{LCP} *occurs at* λ_{max} *and the maximum g-factor at* $\lambda_{g\text{-}factor}$*.*

Au thickness (nm)	λ_{max} (nm)	T_{LCP}	T_{flat}	ΔT	g-factor	$\lambda_{g\text{-}factor}$ (nm)
**27.5**	744	0.458	0.159	0.167	0.45	745
**40.6**	703	0.435	0.072	0.276	0.77	698
**63.5**	684	0.225	0.016	0.158	0.62	670
**79.8**	688	0.128	0.0047	0.084	0.53	667

8.3. Versatility of the Fabrication Method: Lattice Parameter and other Metals (Silver)

The presented fabrication methodology offers a high degree of versatility, enabling the production of metasurfaces with diverse lattice parameters and the deposition of metallic films composed of various materials. This adaptability is exemplified in Figure S9A, which showcases experimental results for a metasurface fabricated with silver (Ag). This Ag metasurface features a film thickness of 33 nm, a lattice parameter of 500 nm, and an average pyramid size of approximately 380 nm.

A key observation is the significant shift in the spectral region exhibiting distinct chiral features towards shorter wavelengths compared to the gold metasurfaces discussed previously. The wavelengths corresponding to the maxima of ΔT and g-factor fall slightly below 600 nm. This finding clearly demonstrates the capability of this methodology to yield structures with chiral activity across the visible spectrum. Notably, the observed ΔT reaches a value of 22.3%, while the g-factor achieves a value of 0.65, both at a wavelength (λ) of 585 nm (Figure S9B). Additionally, a pronounced increase in the g-factor is evident at shorter wavelengths, coinciding with the second-order diffraction of the lattice (λ = 507 nm).

The calculated transmittance spectra and g-factors exhibit excellent agreement with the experimental results (Figure S9C, D). Furthermore, the experimental trend of the mismatch angle (θ_m) observed here aligns well with the trend presented in Figure 4B.

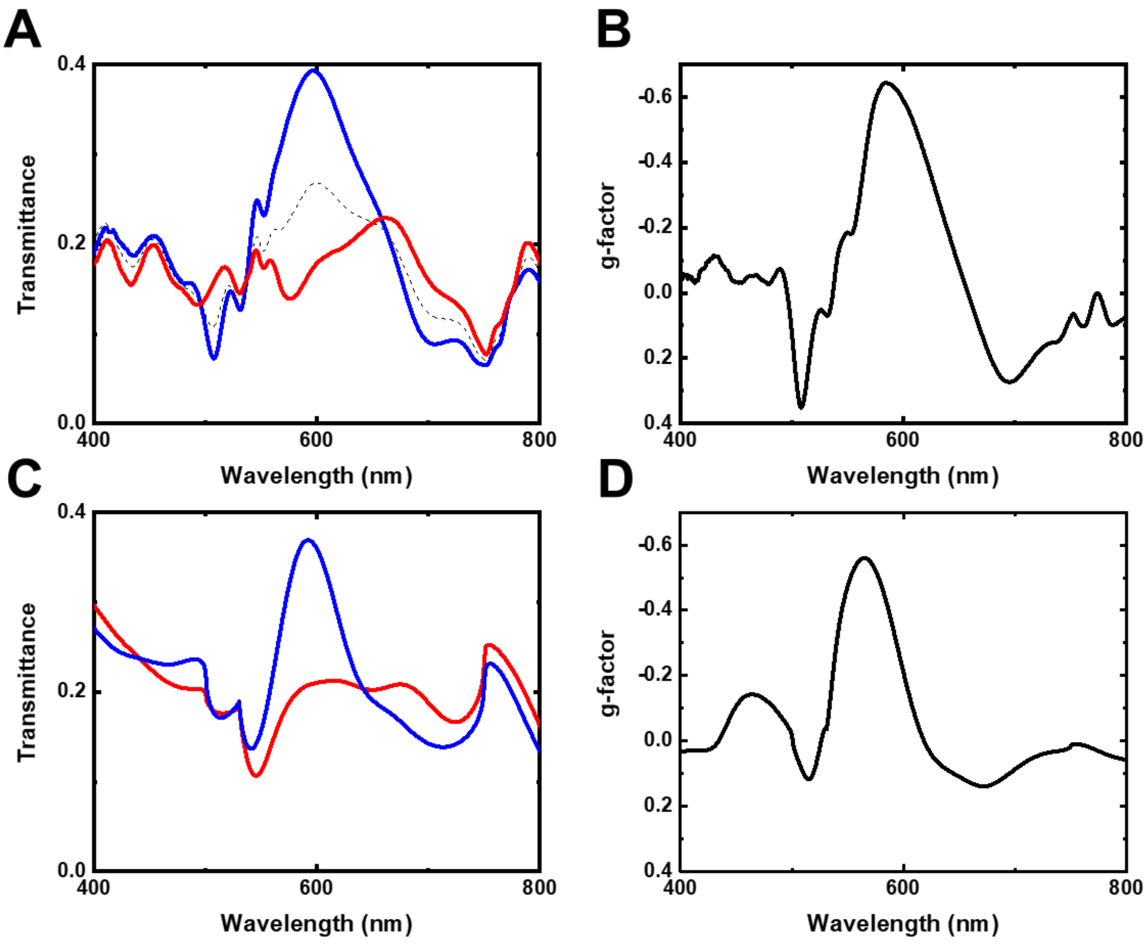

Figure S9 *Influence of metal species and lattice parameter on metasurfaces properties. (A) Experimental transmittance spectra at normal incidence of plasmonic chiral metasurface with a LP of 500 nm, coated with 33nm of Ag. The red and blue lines represent the transmittance for LCP and RCP light, respectively. (B) g-factor spectra extracted from the data presented in panel A. (C, D) FDTD modeled transmittance spectra and g factor.*

9. C/C_0 Dependence on the Mismatch Angle θ_m

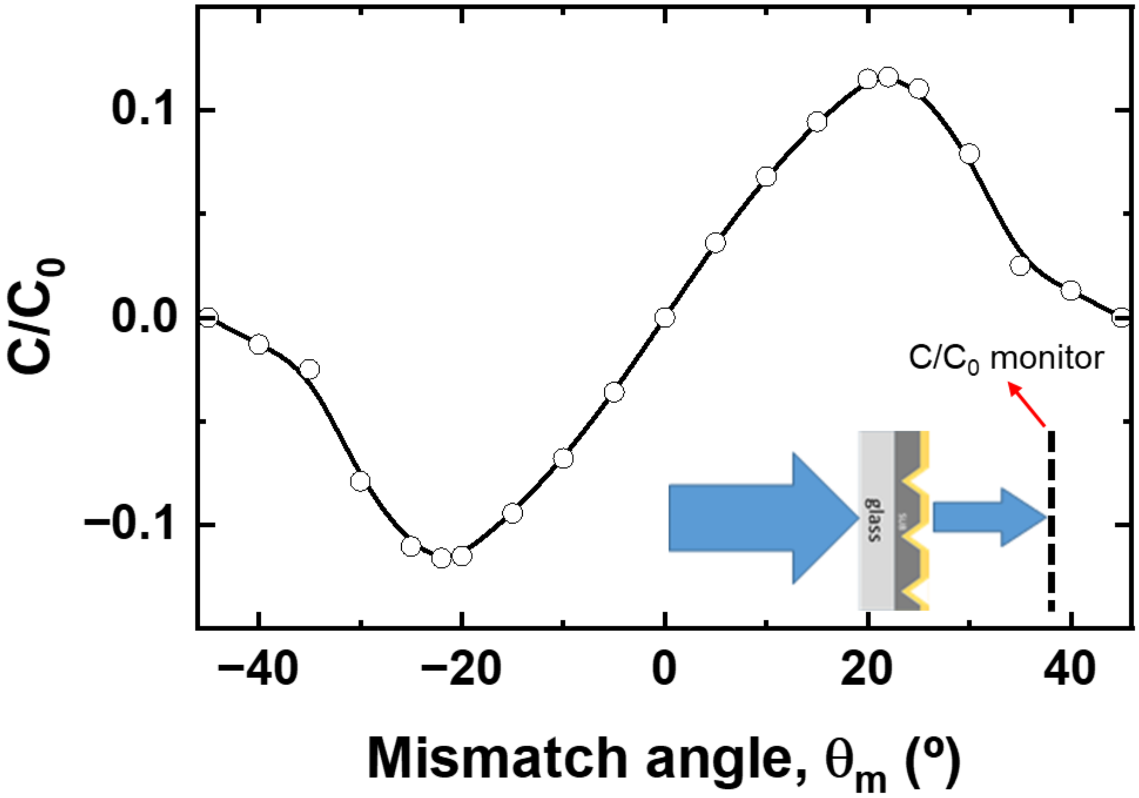

Figure S10 *Dependence of the C-factor on mismatch angle. The C-factor, a measure of the degree of circular polarization, is plotted as a function of the mismatch angle (θ_m). The C factor was computed based on the spatial distributions of $\vec{E}$ and $\vec{H}$ at a monitor positioned 250 nm from the sample (as indicated in the inset schematic configuration).*

10. Additional Information: Temporal Reversibility and Complete Polarimetric Characterization

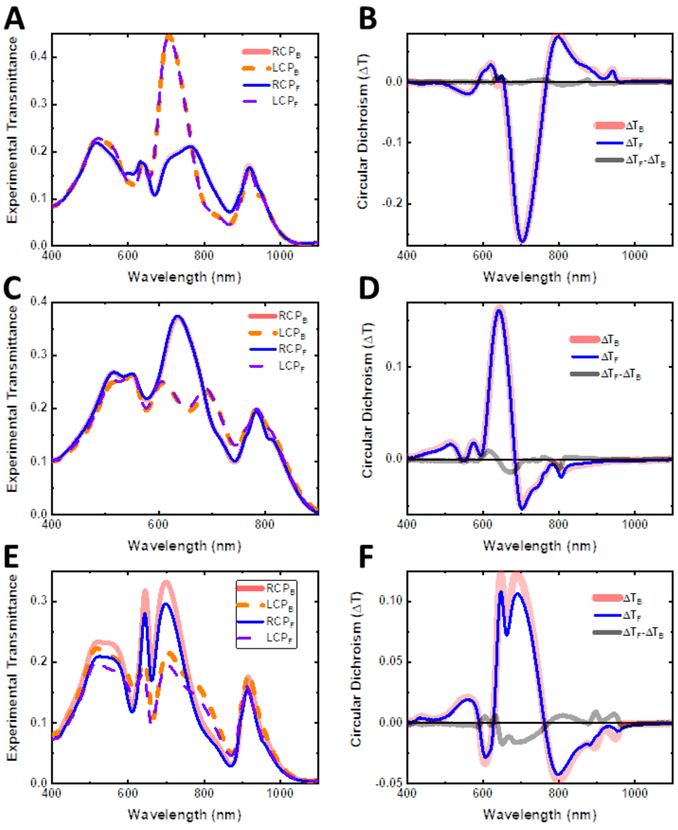

Figure S11 *Experimental transmittance and circular dichroism spectra at forward (from air/metal interface) and backward (air/glass interface) propagation direction for plasmonic chiral metasurfaces with different mismatch angles: (A, B) -23º, (C, D) 16º (E, F) 7º. (A, C, E) RCP (solid lines) and LCP (dashed lines). (B, D, F) ΔT spectra for each propagation direction blue line (forward) and pink line (backward). The grey line displays the difference* ΔT_F - ΔT_B.

10.1. Dependence of the Transmittance Spectra on the Sample Azimuthal Angle

Figure S12 presents the extinction spectra (-log(T)) of the chiral inverted pyramid metasurfaces for LCP, RCP, and linearly polarized light at various azimuthal orientations of the sample. The extinction spectra quantify the light absorbed or scattered by the metasurface.

A key observation is the minimal influence of the azimuthal angle on the overall response for both LCP and RCP light. This indicates that the metasurfaces exhibit similar light extinction characteristics regardless of their in-plane rotation.

In contrast, the spectra for linearly polarized light display a weak dependence on the azimuthal angle across most of the wavelength range. However, at specific wavelengths corresponding to the

first and second Rayleigh anomalies (RAs) (around 900-950 nm and 600-650 nm, respectively), more pronounced variations are observed. These findings suggest that the interaction of linearly polarized light with the metasurface structure exhibits a slight sensitivity to the azimuthal orientation, particularly at the resonant wavelengths.

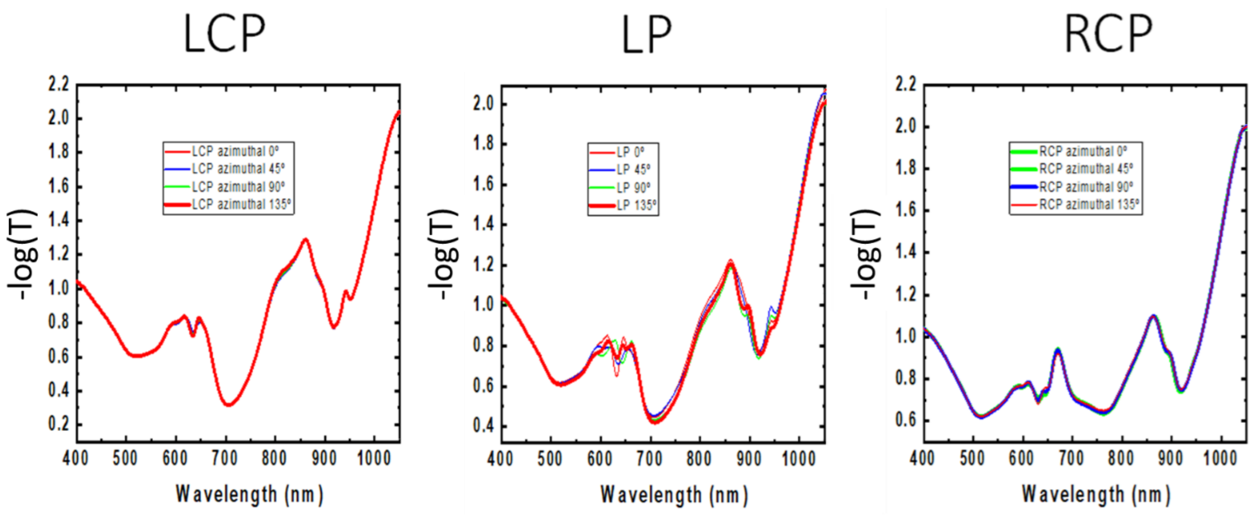

Figure S12 *Extinction spectra for different azimuthal angles of a gold-coated chiral inverted pyramid metasurface. Extinction spectra (-log(T)) are presented for a gold-coated chiral inverted pyramid metasurface under illumination with LCP, linearly polarized, and RCP light at various azimuthal angles.*

10.2. Mueller Matrix Characterization of Chiral Inverted Pyramid Arrays

A custom-built 4-photoelastic modulator (PEM) Mueller matrix polarimeter was employed for the characterization of the metasurfaces in transmission mode.[7] The measurements covered a spectral range of 400-800 nm with a resolution of 2 nm, and the incident light beam diameter was set to 1.5 mm. The obtained Mueller matrix was then utilized to calculate the following key parameters following an analytic calculus:[8, 9]

Linear birefringence (LB): This parameter quantifies the phase shift difference experienced by two orthogonal linearly polarized light axes within the metasurface.

$$2\pi\left(n_x - n_y\right) l/\lambda_0$$

Linear dichroism (LD): This parameter describes the preferential absorption of linearly polarized light with a specific orientation by the metasurface.

$$2\pi\left(\kappa_x - \kappa_y\right) l/\lambda_0$$

Circular birefringence (CB): This parameter quantifies the phase shift difference experienced by LCP and RCP light propagating through the metasurface.

$$2\pi(n_{-} - n_{+})\, l/\lambda_0$$

Circular dichroism (CD): This parameter describes the preferential absorption of LCP or RCP light by the metasurface.

$$2\pi(\kappa_{-} - \kappa_{+})\, l/\lambda_0$$

Where n is the refractive index, κ is the extinction coefficient, l, path length through the medium, λ_0 is the vacuum wavelength of light. Subscripts indicate the polarization direction of light as x in the x axis direction, y in the y axis direction, + circular right, or - circular left.[9, 10]

Note that with the symbols *LB* and *LD* we indicate the overall values of linear birefringence and linear dichroism not just their horizontal projections, that are sometimes denoted by the same symbols. [8]

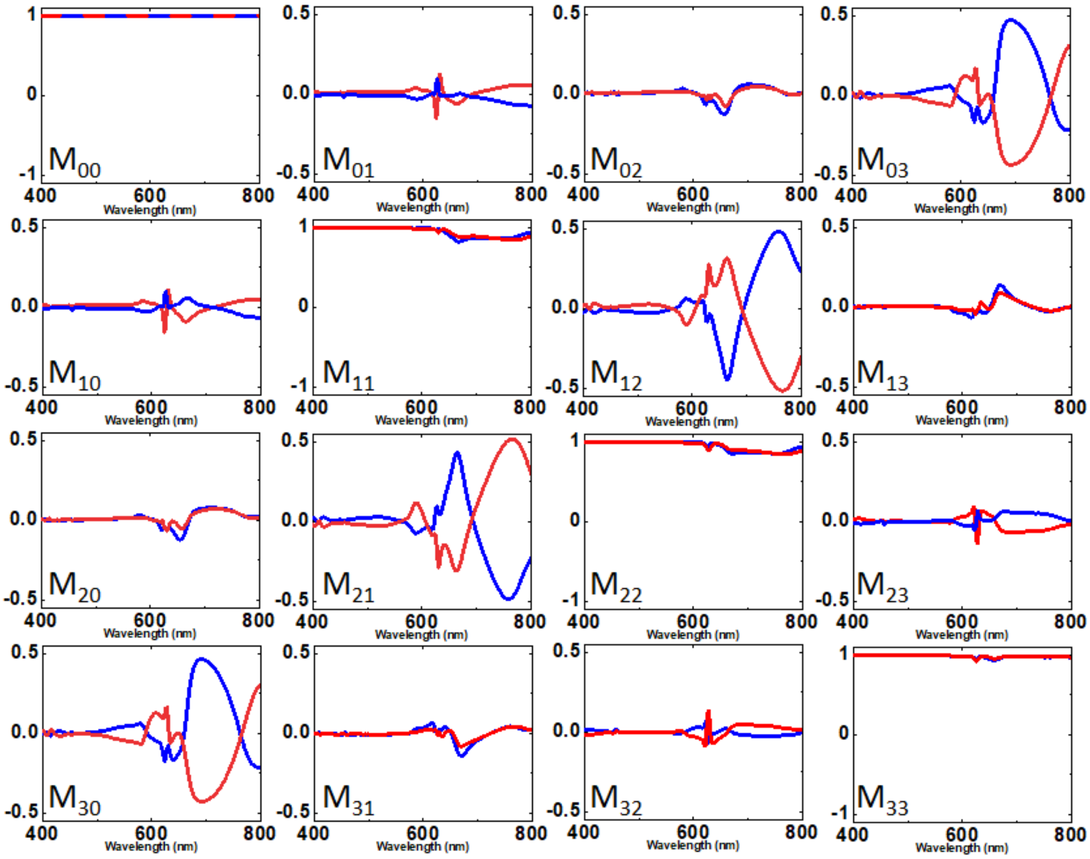

Figure S13 *Experimental Mueller matrix measured in chiral inverted pyramid arrays for two enantiomorphs (blue and red lines) with θ_m +24 and -23, LP = 600 nm, Au layer 40.6 nm.*

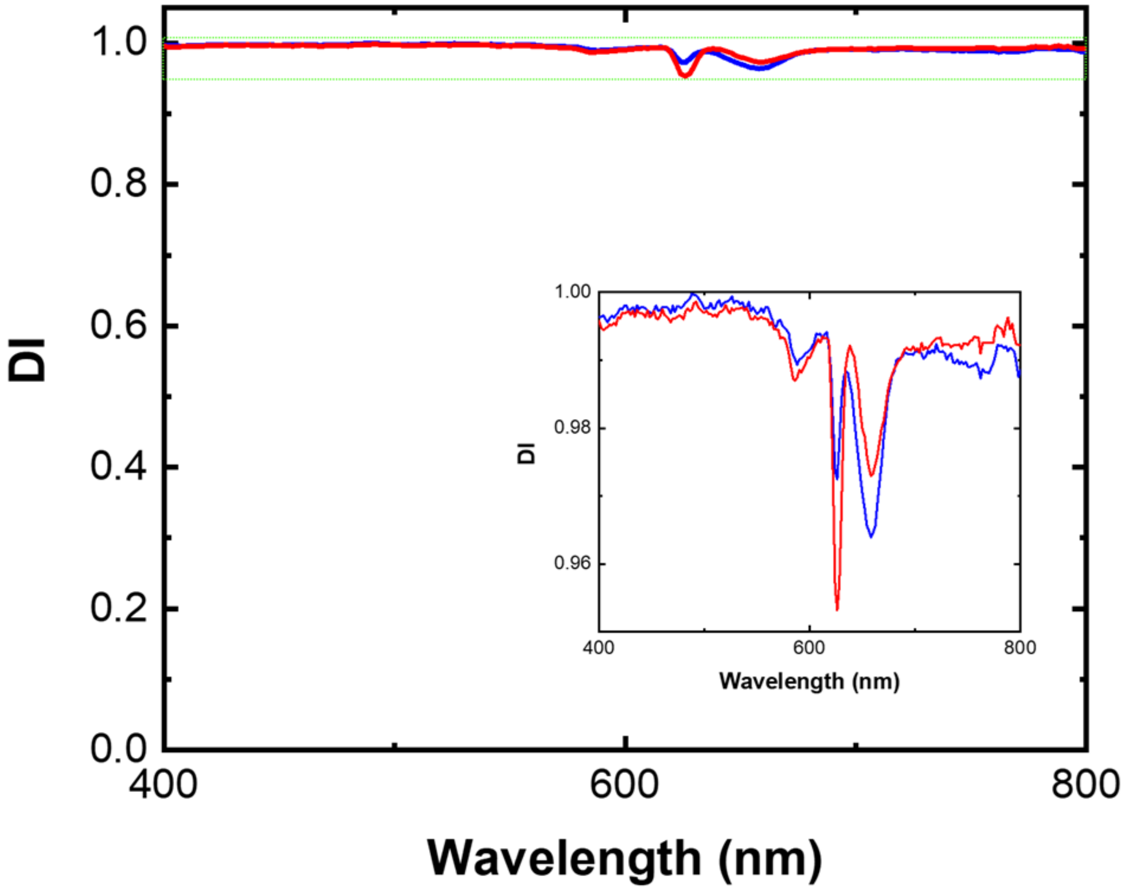

Figure S14 *Experimental Depolarization index calculated from Mueller matrix measured in chiral inverted pyramid arrays for two enantiomorphs (blue and red lines)) with θ_m +24 and -23, LP = 600 nm, Au layer 40.6 nm. The inset shows a zoomed view of the region within the green rectangle.*

11. Chirality Mechanisms in Inverted Pyramid Arrays Metasurfaces

When reviewing the literature on plasmon chirality, a plethora of metrics can be found to quantify the chirality of various systems. These metrics encompass parameters such as ΔT, ΔT/T, T_{LCP}/T_{RCP}, among others, each serving as a distinctive gauge of chirality.[6, 11-15]

Historically, chirality has been extensively used in the study of molecular systems, encompassing both small molecules and macromolecules like proteins.[16] The dissymmetry factor has been a key parameter in quantifying the chirality of molecular systems. The g-factor reflects the differential absorption of LCP and RCP light relative to unpolarized light (Equation 2):

$$g_{factor} = (Abs_{LCP} - Abs_{RCP})/Abs_{nonpol} \quad (2)$$

where Abs_{LCP}, Abs_{RCP}, and Abs_{nonpol} represent the absorbance for LCP, RCP, and non-polarized light, respectively.[17] Using the Lambert-Beer Law, absorbance (extinction) can be expressed as $Abs = \varepsilon b[\,]$ where ε is the extinction coefficient, $\boldsymbol{b}$ is the path length, and [ ] is the concentration of the analyte under study. Since $Abs_{nonpol} = (Abs_{LCP} + Abs_{RCP})/2$, the g-factor can be rewritten as:

$$g_{factor} = \frac{\varepsilon_{LCP} b[\,] - \varepsilon_{RCP} b[\,]}{\varepsilon_{nonpol} b[\,]} = 2\frac{\varepsilon_{LCP} - \varepsilon_{RCP}}{\varepsilon_{LCP} + \varepsilon_{RCP}} \tag{3}$$

The g-factor advantage lies in its dependence solely on the extinction coefficients for LCP and RCP light. This allows its application to particulate systems, a rapidly growing research area (Refs. S18-S20).[18-20]

In the context of colloidal nanoparticles,[21] extinction arises from both absorption (ε_{abs}) and scattering (ε_{sca}) components (ε_{ext} = ε_{abs} + ε_{sca}). In contrast, for molecules where scattering is negligible compared to absorption ($\varepsilon_{abs} \gg \varepsilon_{sca}$), the terms "extinction" and "absorbance" are often interchangeable. However, in colloidal systems, scattering can dominate absorption especially in the case of large colloids, which may exhibits pronounced chiral activity. Therefore, chiral behavior in plasmonic colloids may be primarily due to differential scattering rather than absorption.[22]

For ordered metasurfaces, attenuation in ballistic transmission involves absorption, reflection, and diffraction. Consequently, the g-factor expression requires incorporating these parameters:

$$\boldsymbol{g_{factor} = 2\frac{\Delta\varepsilon_{abs\,L-R} + \Delta\varepsilon_{reflex\,L-R} + \Delta\varepsilon_{diff\,L-R}}{\varepsilon_{LCP} + \varepsilon_{RCP}}} \tag{4}$$

where $\boldsymbol{\Delta\varepsilon_{abs\,L-R}}$, $\boldsymbol{\Delta\varepsilon_{reflex\,L-R}}$, $\boldsymbol{\Delta\varepsilon_{diff\,L-R}}$ represent the difference between LCP and RCP components for absorption, reflection, and diffraction, respectively. For C4-symmetry geometries, zeroth-order reflection is identical for both polarizations, simplifying the equation to

$$\boldsymbol{g_{factor,C_4} = 2\frac{\Delta\varepsilon_{abs\,L-R} + \Delta\varepsilon_{diff\,L-R}}{\varepsilon_{LCP} + \varepsilon_{RCP}}} \tag{5}$$

We can thus conclude that the chiral activity in these structures arises from two contributions:

1. Differential absorption ($\boldsymbol{\Delta\varepsilon_{abs\,L-R}}$), particularly relevant for applications like chiral light photosensors.[23, 24]

2. The lattice capacity to selectively couple specific polarizations $(\boldsymbol{\Delta\varepsilon_{diff\,L-R}})$, resulting in light splitting (differential transmission and diffraction).[25]

These mechanisms combine cumulatively, potentially leading to synergistic or opposing effects. Notably, differential absorption is wavelength-independent (local effect), while the diffraction component is significant only at wavelengths shorter than the Rayleigh anomaly.

11.1. Deconstructing the Chiral Inverted Pyramid Architecture

To gain a deeper understanding of the chiral response of this architecture and its underlying mechanisms, the structural configuration was systematically decomposed into two individual components: the "upper frame" (Φ) and the inverted pyramid (Π) (Figure S15).

Upper Frame (Φ) Analysis

The calculated reflectance (Figure S15 A, D) and transmittance (Figure S15 C, F) spectra for the isolated upper frame (Φ) (Figure S15 B, E) reveal a morphology resembling gammadion-type chiral structures. However, these nanostructures are interconnected, forming a unique fused configuration.

Interestingly, no significant chiral response is observed for the isolated upper frame (Φ) at wavelengths exceeding the λ_{RA} (Figure S15 A-C), consistent with previous reports.[26] This aligns with the absence of diffractive effects at these longer wavelengths.

A clear transition occurs at wavelengths shorter than λ_{RA}. In this regime, collective lattice effects become prominent, leading to the emergence of diffraction-assisted chiral activity. This phenomenon is further amplified by the presence of a substrate (introducing z-axis symmetry breaking), extending the spectral range of significant chiral response beyond 900 nm (Figure S15 D-F).

The inset of Figure S15F highlights an additional effect related to symmetry breaking: differential absorption at wavelengths even longer than λ_{RA}. While the observed difference in absorbance is modest (around 1%), the spectral range extends beyond the conventional diffraction region. This suggests that symmetry breaking can significantly alter the sample's absorption properties.[27]

Inverted Pyramid Array (Π) Analysis

A hypothetical arrangement consisting of an array of unconnected inverted pyramids (Π) was also analyzed (Figure S15 G-L). Unlike the upper frame (Φ), this configuration exhibits chiroactivity not only mediated by diffraction but also at wavelengths exceeding λ_{RA} (Figure S15 I). Notably, the observed transmittance remains independent of light propagation direction, confirming its temporal reversibility.

Similar to the upper frame, incorporating a substrate with the inverted pyramid array (Π) leads to an expansion of the spectral region exhibiting significant diffraction (and differential diffraction) (Figure S15 J-L).

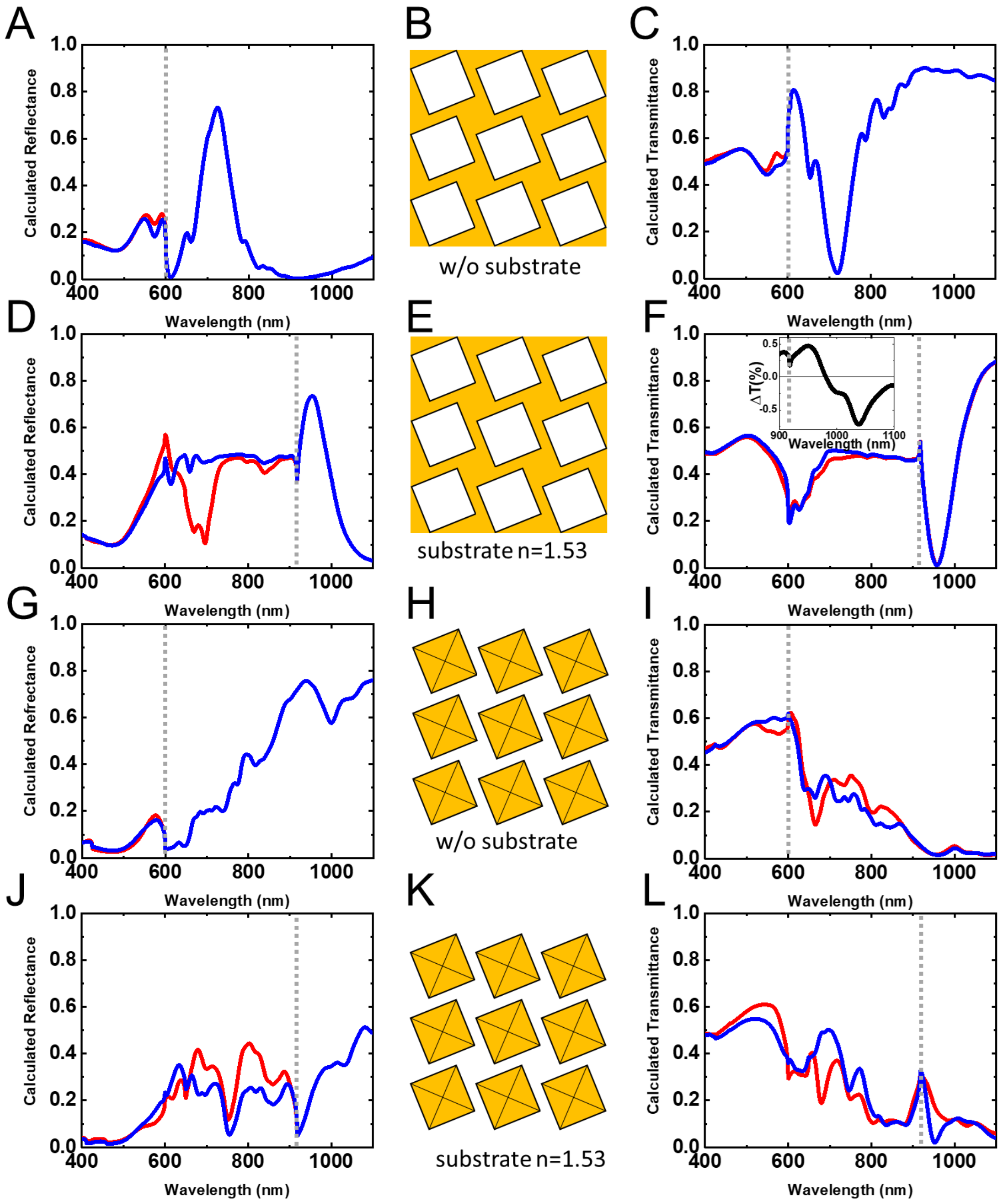

Figure S15 *Calculated reflectance and transmittance spectra at normal incidence under LCP (blue) and RCP (red lines) light, for chiral metasurfaces. Structure Φ a layer with tilted square holes (A-F)) and Π an array of tilted unconnected inverted pyramids (G-L). A-C and G-I, are hypothetical free standing structures without substrate (homogeneous surrounding refractive index = 1). Whereas, D-F and J-L are supported on top of a substrate with n=1.53. The dashed lines represent* λ_{RA}.

11.2. Additional Optical Properties Calculations

Figure S16 presents comprehensive calculations of the optical properties for the investigated structures across various dielectric environments. These calculations explore different substrate and superstrate materials, as well as the effects of inverting the light propagation direction.

A key characteristic of these structures is their reflectance spectra at wavelengths exceeding the λ_{RA}, the reflectance profiles are identical for both circularly polarized polarizations (Figure S16 A, G, M, S, Y). This indicates minimal influence of handedness on reflection spectra outside the diffractive region. Furthermore, even within the diffraction regime, the zeroth-order reflectance spectra for LCP and RCP light remain indistinguishable (Figure S16 B, H, N, T, Z). Therefore, the observed asymmetry in reflectance arises from the differential coupling of light with the lattice based on its polarization state.

To gain further insights, calculations for suspended structures (embedded in a medium with n = 1, representing air) were performed (Figure S16 S-AD). Due to the square lattice geometry with a 600 nm lattice parameter, diffraction is expected only at wavelengths shorter than 600 nm. As anticipated, the calculated reflectance spectra for both LCP and RCP light exhibit identical profiles at wavelengths exceeding 600 nm (Figure S16).

However, the transmittance spectra reveal a critical observation. A peak is observed near 700 nm for both polarizations, but with a significant difference in magnitude: 50% for RCP and 35% for LCP (Figure S16 U-X, AA-AC). This finding highlights a key aspect of the chiral properties. Even without diffraction effects, a substantial difference in light transmittance persists. This suggests that a selective absorption process based on polarization state takes place.

Furthermore, the calculated transmittance spectra for the structure illuminated from both sides show negligible difference, confirming its temporal reversibility. This observation, coupled with the observed polarization-dependent diffraction and selective absorption, strongly suggests that the metasurfaces composed of inverted pyramid arrays with a specific mismatch angle possess intrinsic 3D chirality. These structures function as polarization filters, with selective diffraction in the diffractive regime and selective absorption at wavelengths exceeding $\lambda_{RA.}$

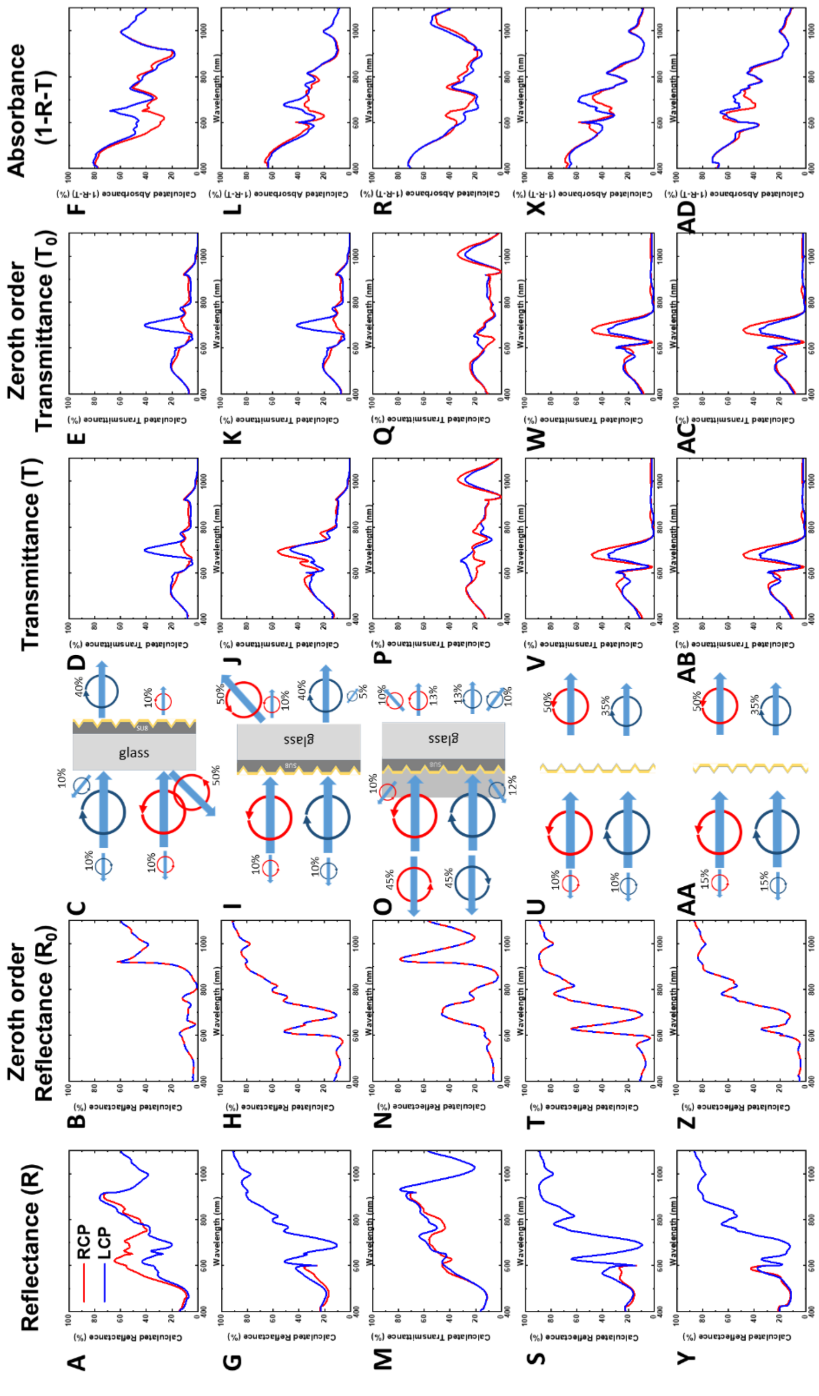

Figure S16 *A compendium of electrodynamic calculations of the optical properties for inverted pyramid arrays with a mismatch angle of -24º, a lattice parameter of 600 nm, and a gold film thickness of 50 nm. (A-F) Supported on a glass substrate, forward propagation direction. (G-L) Supported on a glass substrate, backward propagation direction. (M-R) Index-matched structure (n = 1.53). (S-X) Isolated gold film, backward propagation direction. (Y-AD): Isolated gold film, forward propagation direction. (A, G, M, S, Y) Reflectance (R). (B, H, N, T, Z) Zeroth-order reflectance (R_0). (C, I, O, U, AA) Schematic representation of the calculated structures. (D, J, P, V, AB) Transmittance (T). (E, K, Q, W, AC) Zeroth-order transmittance (T_0). (F, L, R, X, AD) Absorbance spectra (1-R-T).*

11.3. Unveiling the Diffraction-Assisted Chiral Response

Figure S17 delves into the calculated far-field optical properties of the inverted pyramid metasurfaces, focusing on the distribution of diffracted light intensities across various lattice modes. The analysis encompasses zeroth-order diffraction (representing the ballistic component), first-order diffraction modes, and second-order diffraction modes.

Figure S17A and S17B depict the transmittance spectra for LCP and RCP light, respectively, when the light impinges on the sample in the forward direction (air/gold/substrate). Notably, the total transmittance for LCP at 695 nm (56%) is significantly higher than that for RCP (47%).

To delve deeper into the mechanisms responsible for this difference, the transmitted intensity is decomposed into its constituent components: zeroth-order transmittance (T(0,0)), first-order diffraction (T(±1, 0) and T(0, ±1)), and second-order diffraction (T(±1, ±1)). Considering the first and second Rayleigh anomalies relative to the substrate (λ_{RA1} = 915 nm and λ_{RA2} = 650 nm), only two contributions are relevant at the analyzed wavelength (695 nm): ballistic transmittance (T(0,0)) and first-order diffraction (T(±1, 0) and T(0, ±1)) (refer to Figure S18 for angle-resolved transmittance characterization).

For LCP light, a significant portion (46%) is diffracted, while only 10% is transmitted ballistically. Conversely, RCP light exhibits a much higher ballistic transmission (42%) with minimal diffraction (less than 5%). This substantial difference in T(0,0) between the two polarizations is the primary factor contributing to the observed giant circular dichroism (CD) values.

Reversing the light propagation direction simplifies the analysis further. Since the λ_{RA} with respect to air fall at shorter wavelengths, only T(0,0) contributes to the transmittance (Figure S16). Figure S17C and S17D illustrate the spatial distribution of transmitted light for each polarization. For LCP, the intensity of each first-order diffraction mode is comparable to the T(0,0) intensity, whereas for RCP, the light primarily propagates in the ballistic mode with negligible diffraction.

These results demonstrate the remarkable ability of the presented structures to spatially separate the circular components of unpolarized light.[28] This separation involves transmitting one component along the same direction as the incident light, while diffracting the other component at an angle dependent on the wavelength.

In essence, these planar architectures function similarly to a Rochon prism but instead of linearly polarized components it works, splitting unpolarized light into its LCP and RCP components.[29] The interaction with the plasmonic chiral array diffracts one polarization while allowing the other component to pass through undeviated. The combination of optical activity in the visible range, a straightforward fabrication process, and the ability to act as a metasurface beam splitter[25] make these nanoarchitectures highly appealing candidates for various applications.

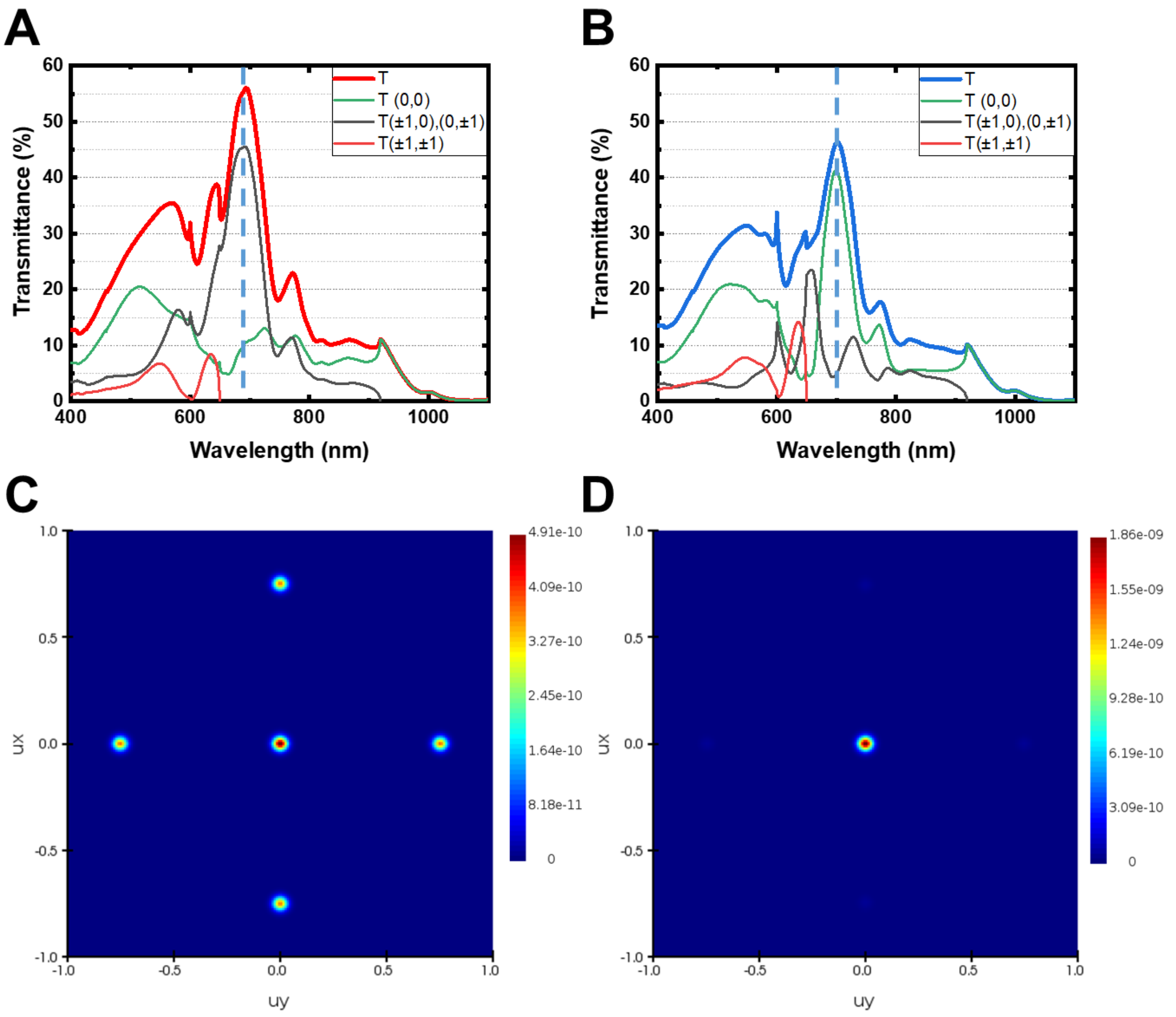

Figure S17 *Calculated total transmittance spectra at normal incidence in forward direction of the plasmonic chiral metasurface. (A) Left circular polarized (LCP) and (B) Right circular polarized (RCP) indicent light. Decomposition in zero-order transmittance (T(0,0), green lines), first diffraction order (T(±1,0), T(0,±1), black lines) and second diffraction order (T(±1, ±1), red lines) are shown in each case. Rayleigh anomalies (λ_{RAs}) relative to the substrate, are λ_{RA1} = 915 nm and λ_{RA2} = 650 nm. (C, D) Far field spatial distribution projection of transmitted light for C) LCP and D) RCP. LP: 600nm, pyramid size: 450 nm, mismatch angle: 22º, Au thickness: 50nm, substrate n=1.53.*

12. Angular Resolved Transmittance Characterization

Transmittance measurements were collected with a custom-built setup consisting of a light source: Halogen Lamp (Ocean Optics, HL-2000-HP, Florida, USA), before the sample, the beam passes through a Glan-Thompson linear polarizer and a superachromatic quarter-wave plate (Thorlabs, SAQWP05M-700, 325–1100 nm) to determine the polarization state of the light; samples were placed on a rotational stage (Thorlabs, RP03/M, New Jersey, USA) to vary the illumination angle

of incidence (AOI) (-45º to +45º with 1º step). Transmitted light was collected by a fiber coupled spectrophotometer (Ocean Insight, QEPro-FL). Air was used as reference.

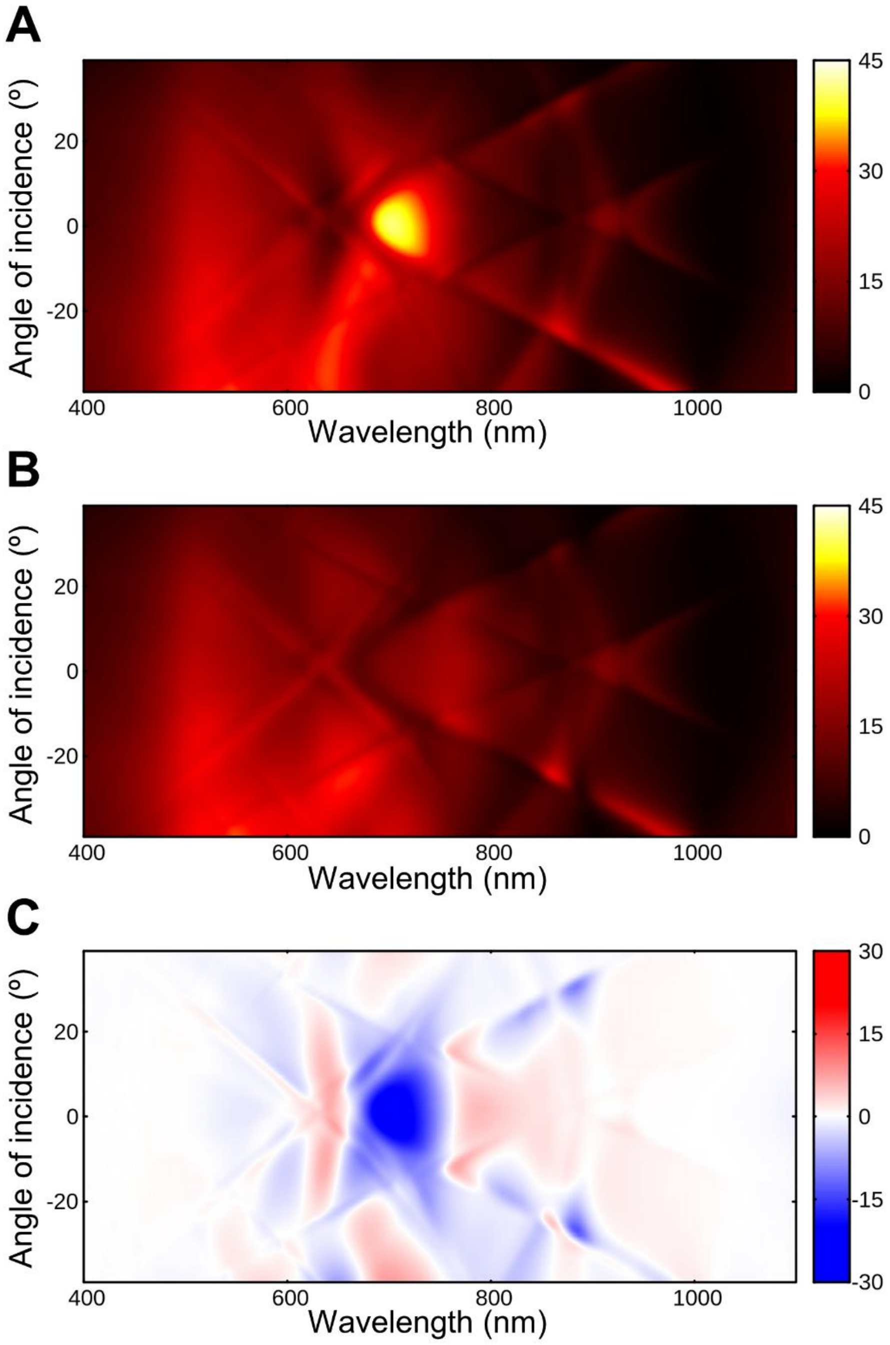

Figure S18 *Chiral inverted pyramid arrays angular-resolved transmittance characterization. Color map for (A) RCP, (B) LCP, (C) ΔT.*

References

(1) Odom, T. W.; Love, J. C.; Wolfe, D. B.; Paul, K. E.; Whitesides, G. M. Improved Pattern Transfer in Soft Lithography Using Composite Stamps. *Langmuir* **2002**, *18* (13), 5314-5320. DOI: 10.1021/la020169l.
(2) Hu, J.; Pérez, L. A.; Garcia-Pomar, J. L.; Mihi, A.; Garriga, M.; Alonso, M. I.; Goñi, A. R. Efficient infrared sunlight absorbers based on gold-covered, inverted silicon pyramid arrays. *Materials Advances* **2022**, *3* (5), 2364-2372, 10.1039/D1MA01237A. DOI: 10.1039/D1MA01237A.
(3) Vaccaro, P. O.; Alonso, M. I.; Garriga, M.; Gutiérrez, J.; Peró, D.; Wagner, M. R.; Reparaz, J. S.; Sotomayor Torres, C. M.; Vidal, X.; Carter, E. A.; et al. Localized thinning for strain concentration in suspended germanium membranes and optical method for precise thickness measurement. *AIP Advances* **2018**, *8* (11), 115131. DOI: 10.1063/1.5050674 (acccessed 6/25/2024).
(4) Kolwas, K. A.-O.; Derkachova, A. Impact of the Interband Transitions in Gold and Silver on the Dynamics of Propagating and Localized Surface Plasmons. LID - 10.3390/nano10071411 [doi] LID - 1411. (2079-4991 (Print)). From 2020 Jul 19.
(5) Qi, X.; Pérez, L. A.; Alonso, M. I.; Mihi, A. High Q-Factor Plasmonic Surface Lattice Resonances in Colloidal Nanoparticle Arrays. *ACS Applied Materials & Interfaces* **2024**, *16* (1), 1259-1267. DOI: 10.1021/acsami.3c08617.
(6) Schäferling, M. *Chiral Nanophotonics*; 2017. DOI: 10.1007/978-3-319-42264-0.
(7) Arteaga, O.; Freudenthal, J.; Wang, B.; Kahr, B. Mueller matrix polarimetry with four photoelastic modulators: theory and calibration. *Applied Optics* **2012**, *51* (28), 6805-6817. DOI: 10.1364/AO.51.006805.
(8) Arteaga, O.; Canillas, A. Analytic inversion of the Mueller–Jones polarization matrices for homogeneous media: erratum. *Optics Letters* **2010**, *35* (20), 3525-3525. DOI: 10.1364/OL.35.003525.
(9) Arteaga, O.; Canillas, A. Analytic inversion of the Mueller-Jones polarization matrices for homogeneous media. *Optics Letters* **2010**, *35* (4), 559-561. DOI: 10.1364/OL.35.000559.
(10) Arteaga, O.; Kahr, B. Mueller matrix polarimetry of bianisotropic materials [Invited]. *Journal of the Optical Society of America B* **2019**, *36* (8), F72-F83. DOI: 10.1364/JOSAB.36.000F72.
(11) Esposito, M.; Tasco, V.; Todisco, F.; Benedetti, A.; Tarantini, I.; Cuscunà, M.; Dominici, L.; De Giorgi, M.; Passaseo, A. Tailoring chiro-optical effects by helical nanowire arrangement. *Nanoscale* **2015**, *7* (43), 18081-18088, 10.1039/C5NR04674B. DOI: 10.1039/C5NR04674B.
(12) Helgert, C.; Pshenay-Severin, E.; Falkner, M.; Menzel, C.; Rockstuhl, C.; Kley, E.-B.; Tünnermann, A.; Lederer, F.; Pertsch, T. Chiral Metamaterial Composed of Three-Dimensional Plasmonic Nanostructures. *Nano Letters* **2011**, *11* (10), 4400-4404. DOI: 10.1021/nl202565e.
(13) Cao, L.; Qi, J.; Wu, Q.; Li, Z.; Wang, R.; Chen, J.; Lu, Y.; Zhao, W.; Yao, J.; Yu, X.; et al. Giant Tunable Circular Dichroism of Large-Area Extrinsic Chiral Metal Nanocrescent Arrays. *Nanoscale Research Letters* **2019**, *14* (1), 388. DOI: 10.1186/s11671-019-3220-7.
(14) Qu, Y.; Bai, Y.; Aba, T.; Ullah, H.; Abudukelimu, A.; Huang, J.; Gou, T.; Li, J.; Zhang, Z. Chiral Near-Fields Induced by Plasmonic Chiral Conic Nanoshell Metallic Nanostructure for Sensitive Biomolecule Detection. *The Journal of Physical Chemistry C* **2020**, *124* (25), 13912-13919. DOI: 10.1021/acs.jpcc.0c03523.
(15) Kilchoer, C.; Abdollahi, N.; Dolan, J. A.; Abdelrahman, D.; Saba, M.; Wiesner, U.; Steiner, U.; Gunkel, I.; Wilts, B. D. Strong Circular Dichroism in Single Gyroid Optical Metamaterials.

Advanced Optical Materials **2020**, *8* (13), 1902131. DOI: https://doi.org/10.1002/adom.201902131 (acccessed 2024/06/28).
(16) Brandt, J. R.; Salerno, F.; Fuchter, M. J. The added value of small-molecule chirality in technological applications. *Nature Reviews Chemistry* **2017**, *1* (6), 0045. DOI: 10.1038/s41570-017-0045.
(17) Long, G.; Adamo, G.; Tian, J.; Klein, M.; Krishnamoorthy, H. N. S.; Feltri, E.; Wang, H.; Soci, C. Perovskite metasurfaces with large superstructural chirality. *Nature Communications* **2022**, *13* (1), 1551. DOI: 10.1038/s41467-022-29253-0.
(18) González-Rubio, G.; Mosquera, J.; Kumar, V.; Pedrazo-Tardajos, A.; Llombart, P.; Solís, D. M.; Lobato, I.; Noya, E. G.; Guerrero-Martínez, A.; Taboada, J. M.; et al. Micelle-directed chiral seeded growth on anisotropic gold nanocrystals. *Science* **2020**, *368* (6498), 1472-1477. DOI: 10.1126/science.aba0980 (acccessed 2024/06/28).
(19) Xu, L.; Wang, X.; Wang, W.; Sun, M.; Choi, W. J.; Kim, J.-Y.; Hao, C.; Li, S.; Qu, A.; Lu, M.; et al. Enantiomer-dependent immunological response to chiral nanoparticles. *Nature* **2022**, *601* (7893), 366-373. DOI: 10.1038/s41586-021-04243-2.
(20) Kim, R. M.; Huh, J.-H.; Yoo, S.; Kim, T. G.; Kim, C.; Kim, H.; Han, J. H.; Cho, N. H.; Lim, Y.-C.; Im, S. W.; et al. Enantioselective sensing by collective circular dichroism. *Nature* **2022**, *612* (7940), 470-476. DOI: 10.1038/s41586-022-05353-1.
(21) Bustamante, C.; Tinoco, I.; Maestre, M. F. Circular differential scattering can be an important part of the circular dichroism of macromolecules. *Proceedings of the National Academy of Sciences* **1983**, *80* (12), 3568-3572. DOI: 10.1073/pnas.80.12.3568 (acccessed 2024/06/28).
(22) Karst, J.; Cho, N. H.; Kim, H.; Lee, H.-E.; Nam, K. T.; Giessen, H.; Hentschel, M. Chiral Scatterometry on Chemically Synthesized Single Plasmonic Nanoparticles. *ACS Nano* **2019**, *13* (8), 8659-8668. DOI: 10.1021/acsnano.9b04046.
(23) Li, W.; Coppens, Z. J.; Besteiro, L. V.; Wang, W.; Govorov, A. O.; Valentine, J. Circularly polarized light detection with hot electrons in chiral plasmonic metamaterials. *Nature Communications* **2015**, *6* (1), 8379. DOI: 10.1038/ncomms9379.
(24) Hou, Y.; Leung, H. M.; Chan, C. T.; Du, J.; Chan, H. L.-W.; Lei, D. Y. Ultrabroadband Optical Superchirality in a 3D Stacked-Patch Plasmonic Metamaterial Designed by Two-Step Glancing Angle Deposition. *Advanced Functional Materials* **2016**, *26* (43), 7807-7816. DOI: https://doi.org/10.1002/adfm.201602800 (acccessed 2024/06/19).
(25) Shen, Z.; Huang, D. A Review on Metasurface Beam Splitters. In *Nanomanufacturing*, 2022; Vol. 2, pp 194-228.
(26) Arteaga, O.; Sancho-Parramon, J.; Nichols, S.; Maoz, B. M.; Canillas, A.; Bosch, S.; Markovich, G.; Kahr, B. Relation between 2D/3D chirality and the appearance of chiroptical effects in real nanostructures. *Optics Express* **2016**, *24* (3), 2242-2252. DOI: 10.1364/OE.24.002242.
(27) Kuwata-Gonokami, M.; Saito, N.; Ino, Y.; Kauranen, M.; Jefimovs, K.; Vallius, T.; Turunen, J.; Svirko, Y. Giant Optical Activity in Quasi-Two-Dimensional Planar Nanostructures. *Physical Review Letters* **2005**, *95* (22), 227401. DOI: 10.1103/PhysRevLett.95.227401.
(28) Khorasaninejad, M.; Crozier, K. B. Silicon nanofin grating as a miniature chirality-distinguishing beam-splitter. *Nature Communications* **2014**, *5* (1), 5386. DOI: 10.1038/ncomms6386.

(29) Wang, B.; Dong, F.; Feng, H.; Yang, D.; Song, Z.; Xu, L.; Chu, W.; Gong, Q.; Li, Y. Rochon-Prism-Like Planar Circularly Polarized Beam Splitters Based on Dielectric Metasurfaces. *ACS Photonics* **2018**, *5* (5), 1660-1664. DOI: 10.1021/acsphotonics.7b01191.
